# Computer-assisted quantification of tumor-associated collagen signatures to improve the prognosis prediction of breast cancer

**DOI:** 10.1186/s12916-021-02146-7

**Published:** 2021-11-18

**Authors:** Gangqin Xi, Lida Qiu, Shuoyu Xu, Wenhui Guo, Fangmeng Fu, Deyong Kang, Liqin Zheng, Jiajia He, Qingyuan Zhang, Lianhuang Li, Chuan Wang, Jianxin Chen

**Affiliations:** 1 Key Laboratory of OptoElectronic Science and Technology for Medicine of Ministry of Education, Fujian Provincial Key Laboratory of Photonics Technology, College of Photonic and Electronic Engineering, Fujian Normal University, Fuzhou, 350007 China; 2 College of Physics and Electronic Information Engineering, Minjiang University, Fuzhou, 350108 China; 3 Department of General Surgery, Nanfang Hospital, Southern Medical University, Guangzhou, 510515 China; 4 Breast Surgery Ward, Department of Breast Surgery, Department of General Surgery, Fujian Medical University Union Hospital, Fuzhou, 350001 China; 5 Department of Pathology, Fujian Medical University Union Hospital, Fuzhou, 350001 China; 6 Department of Medical Oncology, Harbin Medical University Cancer Hospital, Harbin, 150081 China

**Keywords:** Breast cancer, Multiphoton imaging, TACS corresponding microscopic features, Prognosis

## Abstract

**Background:**

Collagen fibers play an important role in tumor initiation, progression, and invasion. Our previous research has already shown that large-scale tumor-associated collagen signatures (TACS) are powerful prognostic biomarkers independent of clinicopathological factors in invasive breast cancer. However, they are observed on a macroscale and are more suitable for identifying high-risk patients. It is necessary to investigate the effect of the corresponding microscopic features of TACS so as to more accurately and comprehensively predict the prognosis of breast cancer patients.

**Methods:**

In this retrospective and multicenter study, we included 942 invasive breast cancer patients in both a training cohort (*n* = 355) and an internal validation cohort (*n* = 334) from one clinical center and in an external validation cohort (*n* = 253) from a different clinical center. TACS corresponding microscopic features (TCMFs) were firstly extracted from multiphoton images for each patient, and then least absolute shrinkage and selection operator (LASSO) regression was applied to select the most robust features to build a TCMF-score. Finally, the Cox proportional hazard regression analysis was used to evaluate the association of TCMF-score with disease-free survival (DFS).

**Results:**

TCMF-score is significantly associated with DFS in univariate Cox proportional hazard regression analysis. After adjusting for clinical variables by multivariate Cox regression analysis, the TCMF-score remains an independent prognostic indicator. Remarkably, the TCMF model performs better than the clinical (CLI) model in the three cohorts and is particularly outstanding in the ER-positive and lower-risk subgroups. By contrast, the TACS model is more suitable for the ER-negative and higher-risk subgroups. When the TACS and TCMF are combined, they could complement each other and perform well in all patients. As expected, the full model (CLI+TCMF+TACS) achieves the best performance (*AUC* 0.905, [0.873–0.938]; 0.896, [0.860–0.931]; 0.882, [0.840–0.925] in the three cohorts).

**Conclusion:**

These results demonstrate that the TCMF-score is an independent prognostic factor for breast cancer, and the increased prognostic performance (TCMF+TACS-score) may help us develop more appropriate treatment protocols.

**Supplementary Information:**

The online version contains supplementary material available at 10.1186/s12916-021-02146-7.

## Background

Most of cancer-related mortality is directly caused by the cancer cell metastasis from the primary tumor to distant sites [1]. The migration of cancer cells is a multistep process and begins with a remodeling of the local tumor microenvironment (TME) including changes in the extracellular matrix (ECM) [2]. The ECM provides structural and mechanical support for cells and tissues, influences cell migration through its physical properties [3, 4], and is mainly made up of a complex meshwork of collagen fibers, glycoproteins, and proteoglycans [5]. Collagen fibers, which are the important component of ECM and may promote or inhibit cell motion, either impede tumor invasion via acting as a barrier against migration [6] or facilitate invasion through providing high-speed “highways” according to their orientation [7]. The role of neighboring stroma in mediating breast tumor initiation, progression, and invasion to surrounding tissue has been broadly researching [8, 9].

There has a famous prognostic factor associated with collagen orientation, known as TACS1-3, which predicts the behavior of cancer cells according to the mode of collagen alignment [10]. In vitro research showed that collagen orientations are crucial to tumor cell invasion [11], and in vivo study also proven that collagen signature is an important prognostic factor [12]. Especially, TACS3, which was identified via the presence of linear collagen structure perpendicular to tumor border, shows an important prognostic value and is related to reducing the survival rate of patients [12]. In our previous research, we expanded the TACS1-3 by recognizing TACS4-8 at a large scale and at the invasion front of the primary tumor. TACS1-8 could describe the changes of collagen distribution patterns caused by the interaction between tumors and surrounding collagen fibers during the process of tumorigenesis, development, and invasion. We found that TACS-score (combining TACS1-8) was a strong and general prognostic indicator for disease-free survival (DFS) of breast cancer patients [13]. However, the microscopic features of TACS have not been systematically investigated.

Many new computational techniques were developed to quantify the collagen microstructures and provided robust and informative features within a heterogeneous collection of collagen patterns [14]. For example, Bredfeldt et al. have applied the curvelet-denoising filter following by the FIRE (CT-FIRE) to track collagen shape changes over time in an in vivo mouse model [15]. Falzon et al. have used the elliptical Fourier analysis to compare the differences in collagen shape between normal, benign, and malignant breast tissues [16], and Hu et al. suggested the orientation-dependent gray-level co-occurrence method (OD-GLCM) for distinguishing the different texture patterns of collagen fibers in rat tendons and human pancreatic tissue [17]. Multiphoton microscopy (MPM) has the talent to provide detailed tissue architecture information of unprocessed specimens through a combination of two-photon excitation fluorescence (TPEF) and second harmonic generation (SHG), where SHG imaging could provide a direct and label-free approach for observing collagen structures. Recently, the feature extraction of SHG images is a rapidly growing field, which involves in extracting numerous quantitative features to determine the relationships between the microscopic features and potential pathology [18, 19]. Nevertheless, the association between collagen microstructure characteristics and breast cancer prognosis has not been fully explored.

In this study, we first converted TACS1-8-related SHG images into high-dimensional minable data and extracted TACS corresponding microscopic features (TCMFs). Then, the least absolute shrinkage and selection operator (LASSO) regression was used for choosing the most robust features to build a TCMF-score. In contrast to the results from TACS, the TCMF-score is more suitable for identifying low-risk patients. Furthermore, when the TACS and TCMF models were combined, they could complement each other and perform well in all patients. Therefore, this strong and general imaging prognosticator (TCMF-score) may convince pathologists to adopt it as a prognostic histopathology tool, thereby broadly altering therapeutic options.

## Methods

### Patients and clinicopathological information

The institutional review board at Fujian Medical University Union Hospital and Harbin Medical University Cancer Hospital provided ethical approval for the use of patient material in this study. We retrospectively collected 1223 formalin-fixed paraffin-embedded (FFPE) breast cancer tissue samples from 1223 patients (age 21–87 years), where 281 samples were excluded according to the exclusion criteria and 942 passed quality control for the final analysis. Samples were included in terms of a consecutive series of predefined inclusion criteria: histologic diagnosis of invasive breast cancer without distant metastasis, more than 5 years of follow-up except for patients who developed distant relapse within 5 years. The exclusion criteria were as follows: patients were treated with preoperative therapy (neoadjuvant chemotherapy or radiotherapy); patients had missed relevant clinicopathological characteristics or follow-up data; some samples were not suitable for analysis due to the damage, tumor-free, or poor section quality; some samples’ collagen fibers are too sparse to extract their microscopic features.

For this study, 689 patients were from the Fujian Medical University Union Hospital and were divided randomly into training (355 cases) and internal validation (334 cases) cohorts. The external validation cohort was comprised of 253 patients from Harbin Medical University Cancer Hospital. Table 1 shows the clinical characteristics of the patients in the three cohorts. The patient recruitment pathway is shown in Additional file 1: Fig. S1. Patients in the three cohorts are balanced for disease-free survival (DFS), with the median DFS time (*IQR*) of 71.0 (38.0–84.0) months for the training cohort, 70.0 (35.8–83.0) months for the internal validation cohort, and 70.0 months (40.5–80.0) for the external validation cohort. Baseline clinical characteristics were collected, including age at surgical intervention, molecular subtype (luminal A and B, HER2-enriched as well as triple-negative), tumor size (T1, T2, and T3), lymph node metastasis (N0, N1, and N2), clinical stage (I, II, and III), histological grade (G1, G2, and G3), chemotherapy (yes or no), radiation therapy (yes or no), endocrine therapy (yes or no), and targeted therapy (yes or no).

**Table 1:** Baseline characteristics of patients in the training, internal validation, and external validation cohorts

Characteristics	Fuzhou training cohort (355)	Fuzhou internal validation cohort (334)	Harbin external validation cohort (253)	Total cohort (942)
**Age**				
≤50	199 (56.1%)	194 (58.1%)	136 (53.8%)	529 (56.2%)
>50	156 (43.9%)	140 (41.9%)	117 (46.2%)	413 (43.8%)
**Molecular subtype**				
Luminal A	75 (21.1%)	59 (17.7%)	69 (27.3%)	203 (21.5%)
Luminal B	161 (45.4%)	145 (43.4%)	97 (38.3%)	403 (42.8%)
HER2-enriched	64 (18.0%)	75 (22.5%)	48 (19.0%)	187 (19.9%)
Triple negative	55 (15.5%)	55 (16.5%)	39 (15.4%)	149 (15.8%)
**Tumor size**				
≤2cm	147 (41.4%)	134 (40.1%)	143 (56.5%)	424 (45.0%)
2–5cm	187 (52.7%)	174 (52.1%)	107 (42.3%)	468 (49.7%)
>5cm	21 (5.9%)	26 (7.8%)	3 (1.2%)	50 (5.3%)
**Nodal status**				
0	180 (50.7%)	169 (50.6%)	117 (46.3%)	466 (49.5%)
1–3	74 (20.8%)	81 (24.3%)	75 (29.6%)	230 (24.4%)
≥4	101 (28.5%)	84 (25.1%)	61 (24.1%)	246 (26.1%)
**Clinical stage**				
I	96 (27.0%)	83 (24.9%)	74 (29.3%)	253 (26.9%)
II	156 (43.9%)	161 (48.2%)	117 (46.2%)	434 (46.1%)
III	103 (29.1%)	90 (26.9%)	62 (24.5%)	255 (27.0%)
**Histological grade**				
G1	56 (15.8%)	56 (16.8%)	11 (4.4%)	123 (13.1%)
G2	194 (54.6%)	176 (52.7%)	202 (79.8%)	572 (60.7%)
G3	105 (29.6%)	102 (30.5%)	40 (15.8%)	247 (26.2%)
**ER**				
Negative	119 (33.5%)	130 (38.9%)	87 (34.4%)	336 (35.7%)
Positive	236 (66.5%)	204 (61.1%)	166 (65.6%)	606 (64.3%)
**PR**				
Negative	152 (42.8%)	155 (46.4%)	108 (42.7%)	415 (44.1%)
Positive	203 (57.2%)	179 (53.6%)	145 (57.3%)	527 (55.9%)
**HER2**				
Negative	243 (68.5%)	213 (63.8%)	171 (67.6%)	627 (66.6%)
Positive	112 (31.5%)	121 (36.2%)	82 (32.4%)	315 (33.4%)
**Chemotherapy**				
No	28 (7.9%)	28 (8.4%)	27 (10.7%)	83 (8.8%)
Yes	327 (92.1%)	306 (91.6%)	226 (89.3%)	859 (91.2%)
**Endocrine therapy**				
No	135 (38.0%)	140 (41.9%)	133 (52.6%)	408 (43.3%)
Yes	220 (62.0%)	194 (58.1%)	120 (47.4%)	534 (56.7%)
**Radiation therapy**				
No	234 (65.9%)	232 (69.5%)	195 (77.1%)	661 (70.2%)
Yes	121 (34.1%)	102 (30.5%)	58 (22.9%)	281 (29.8%)
**Targeted therapy**				
No	331 (93.2%)	311 (93.1%)	228 (90.1%)	870 (92.4%)
Yes	24 (6.8%)	23 (6.9%)	25 (9.9%)	72 (7.6%)

Note: *ER*, estrogen receptor; *PR*, progesterone receptor

### Sample preparation and computer-based quantitation of TACS corresponding microscopic features (TCMFs)

Two serial sections of 5-μm thickness were cut from archived paraffin block via a semiautomatic microtome in the pathology department. One slice was deparaffinized by alcohol and xylene and stained with H&E for whole slide imaging, and the other adjacent section was simply deparaffinized for stain-free MPM imaging.

The protocol for TACS1-8 quantification has been described in detail in a previously published paper [13]. For each case, the FFPE tissue block with tumor boundary was selected by a pathologist for microscopic analysis of collagen features. Throughout the whole tissue slice, several (7–20) discrete ~2.8-mm squared non-overlapping regions across the invasive margin and adjacent tumor areas, which depended on the size of samples, were firstly numbered in H&E images. Then, MPM imaging was performed on another section based on all the numbered regions. The large-scale TACS were determined on the MPM images by three independent reviewers who did not know the pathological outcomes.

Subsequently, for each patient, a region of interest (ROI) with a field of view of 150μm × 150μm was identified from each non-overlapping large-scale MPM image with TACS patterns. A total of 142 TACS corresponding microscopic features (TCMFs), including 8 morphologic features and 134 textural features, were first extracted from each ROI using Matlab 2016b (Additional file 1: Supplementary Methods [20–28], Table S8-S11, Fig. S8, Fig. S10) and averaged over all ROIs from each patient, and then all 142 TCMFs were normalized using *Z*-score transformation. The average time of MPM imaging on a slice (a patient) was about 60 min, and the examination time for a trained reviewer to extract TACS was about 10 min/section. The average time of the intercepting small-scale image and running Matlab program was about 6 min/section.

### Statistical analysis

The statistical analysis was performed on R 3.5.2 and IBM SPSS Statistics 24. The clinical endpoint of our study was disease-free survival (DFS) that was defined as the time from the date of diagnosis to that of the first recurrence of the disease, date of death, date last known to have no evidence of disease, or date of the most recent follow-up. All statistical tests were two-sided, and a *P* < 0.05 was considered statistically significant.

The least absolute shrinkage and selection operator (LASSO) regression was applied to choose the most robust features to establish a TCMF-score. LASSO is a popular regression method for the high-dimensional predictors [29] and has been expanded to the Cox proportional hazard regression model for survival analysis [30]. The R package “glmnet” was selected to implement the LASSO Cox regression model analysis [31]. We applied the LASSO algorithm jointly with the Cox survival model to perform a nested feature selection scheme to analyze the association between each TCMF feature and DFS in the training cohort. A formula was obtained through a linear combination of the selected features weighted by their respective LASSO coefficients and then was applied to acquire a TCMF-score for each patient. We used multivariate Cox regression analysis to calculate the relative weight of each score (TCMF-score, TACS-score, CLI-score) and then used a linear combination of each score and its relative weight to establish a comprehensive prognosis score (TCMF+TACS or CLI+TCMF+TACS).

We performed a receiver operating characteristic (ROC) curve analysis to obtain the areas under the curves (*AUC*s) for estimating prognostic accuracy and to determine the optimal cutoff value by maximizing the Youden index in the training cohort. Then, the cutoff value was applied to classify the patients into the low- and high-risk groups. The Kaplan-Meier survival curves were used for evaluating the correlation between variables and disease-free survival, and the log-rank test was used to analyze the differences in survival between the two groups. Univariate and multivariate Cox proportional hazard regression analysis was used for choosing independent predictors by likelihood ratio test [32], and then we made use of the independent predictors to establish the nomogram and generate a comprehensive indicator for assessing DFS. The performance of the nomogram was evaluated via discrimination and calibration [33]. A concordance index (C-index) which ranged from 0.5 (no discrimination at all) to 1.0 (perfect discrimination) was used for estimating the discrimination, and a calibration plot, which was a graphic representation of the relationship between the actual incidence and predicted probabilities, was used for evaluating the calibration. A calibration curve would be close to the 45° diagonal line for a well-calibrated model [34].

## Results

### TACS corresponding microscopic feature score (TCMF-score)

**Fig. 1:**
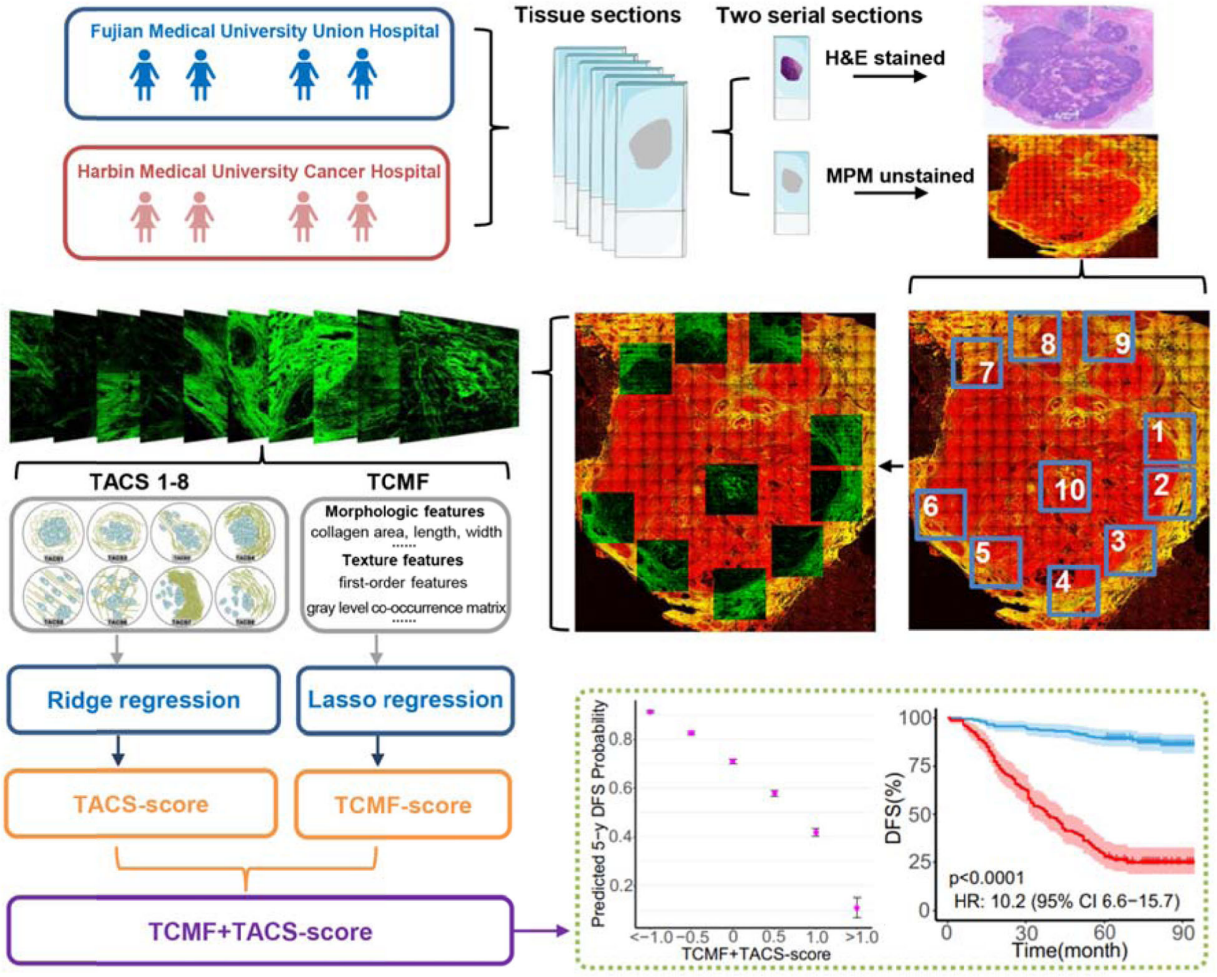
The flowchart of this study. Large-scale TACSs were visually examined on SHG images by three independent reviewers. A total of 142 TACS corresponding microscopic features (TCMFs) were extracted from SHG images. Ridge regression and LASSO regression were used to calculate the TACS- and TCFM-score, and then the two scores were combined for a series of prognostic analysis. TACS1, curved collagen fibers wrap around the emergent tumor foci; TACS2, collagen fibers are stretched due to tumor growth and align more parallel to tumor boundary; TACS3, collagen fibers align perpendicular to the tumor boundary in a radiation pattern to facilitate tumor cell migration; TACS4, reticular distribution of collagen fibers adjacent to expanding tumor that leads to a clear tumor boundary; TACS5, directionally distributed collagen fibers that enable unidirectional tumor cell migration without a clear tumor boundary; TACS6, chaotically aligned collagen fibers that enable multidirectional tumor cell migration without a clear tumor boundary; TACS7, densely distributed collagen fibers at the tumor invasion front largely free of tumor cells; TACS8, sparsely distributed collagen fibers at the tumor invasion front largely free of tumor cells

Our previous results suggested that the TACS-score is a key determinant of invasive breast cancer prognosis. We recognized 8 major TACSs in the large-scale MPM images, in a way similar to identify biomarkers of histopathological subtypes from H&E images. TACS1-8 were mainly based on the macroscopic appearance of collagen morphological changes in the tumor microenvironment. Details of the TACS-score for each patient have been presented previously [13]. The calculation formula of the TACS-score was in Additional file 1: Supplementary Methods. In this study, the TACS-score is a comprehensive prognostic manifestation of TACS1-8, namely TACS1-8-score. All TACS-score description in the text refers to the TACS1-8-score and TACS is the abbreviation for TACS1-8. After obtaining the macrostructural information by MPM images, we extracted the corresponding microscopic features (TCMF) of these 8 patterns. The SHG image was segmented and 142 features were extracted. The flowchart of this study is shown in Fig. 1. Not all of the aforementioned 142 features are associated with prognosis, and irrelevant or redundant features might potentially lower the prediction quality of the predictor. High-dimensional feature selection could remove irrelevant features and retain the most relevant features for building a predictive model, and there can contribute to lifting the efficiency of learning tasks as well as making the model easier to be understood. In this study, we used the least absolute shrinkage and selection operator (LASSO) to capture 14 robust microscopic features associated with prognosis. A detailed description of the microscopic collagen features, selection of the final features, and the formula for calculating the TCMF-score is shown in Additional file 1: Supplementary Methods and Fig. S7.

**Fig. 2:**
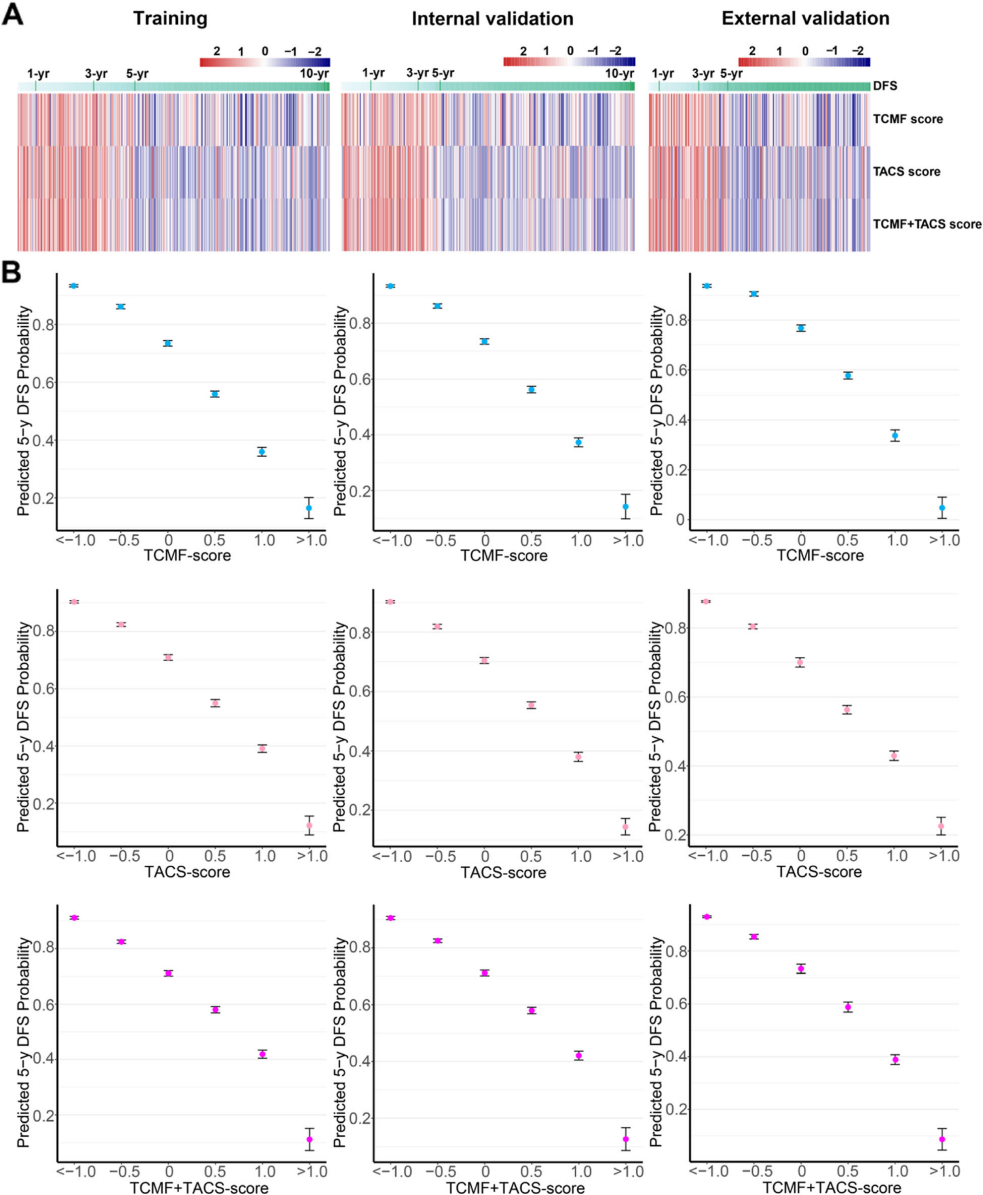
**A** Heatmaps show the relationship between the TCMF-score, TACS-score, and TCMF+TACS-score of the three cohorts and disease-free survival (DFS). **B** The 5-year DFS probability is predicted based on the TCMF-score, TACS-score, and TCMF+TACS-score in the three cohorts

As shown in Fig. 2A, the color bar of the heatmap indicates the relationship between the risk scores and DFS. A lower score (TCMF-score, TACS-score, or TCMF+TACS-score) is associated with better prognosis (higher 5-year DFS), while a higher score is associated with worse prognosis (lower 5-year DFS). Figure 2A also shows that there is a relatively apparent demarcation line at 5 years for the three scores in the three cohorts. Most of the bars larger than 5 years are blue and have a good prognosis, and most of the bars lower than 5 years are red and have a poor prognosis. In the training cohort, the lower TCMF-score, TACS-score, and TCMF+TACS-score (<−1) predict higher 5-year DFS rate (93.3%, *CI*, 92.9–93.7%; 90.3%, *CI*, 89.9–90.8%; 91.1%, *CI*, 90.6–91.7%), and the higher score (>1) indicated lower 5-year DFS rate (16.5%, *CI*, 12.6–20.4%; 12.2%, *CI*, 8.8–15.5%; 11.2%, *CI*, 7.1–15.3%). Similar findings were also obtained in the validation cohorts. The probability of 5-year DFS predicted by the scores is presented in Fig. 2B. The histograms of the three scores can be seen in the supplementary material (Additional file 1: Fig. S2) and are evenly distributed in the three cohorts. This scoring system may act as an auxiliary tool for pathologists to estimate patients’ survival.

### Predictive performance of the TCMF-score

The TCMF-score is significantly associated with DFS in univariate Cox proportional hazard regression analysis (*HR* 3.667, [2.716–4.953], *P* < 0.0001). After adjusting for clinical variables by multivariate Cox regression analysis, the TCMF-score remains an independent prognostic indicator for predicting DFS (*HR* 1.911, [1.407–2.595], *P* < 0.0001) (Additional file 1: Table S1) in the training cohort. Noteworthy, collagen signatures remain to be the most significant factors (with the smallest *P* values in all cohorts) compared to the clinicopathological factors in the other validation cohorts (Additional file 1: Table S2, S3).

**Fig. 3:**
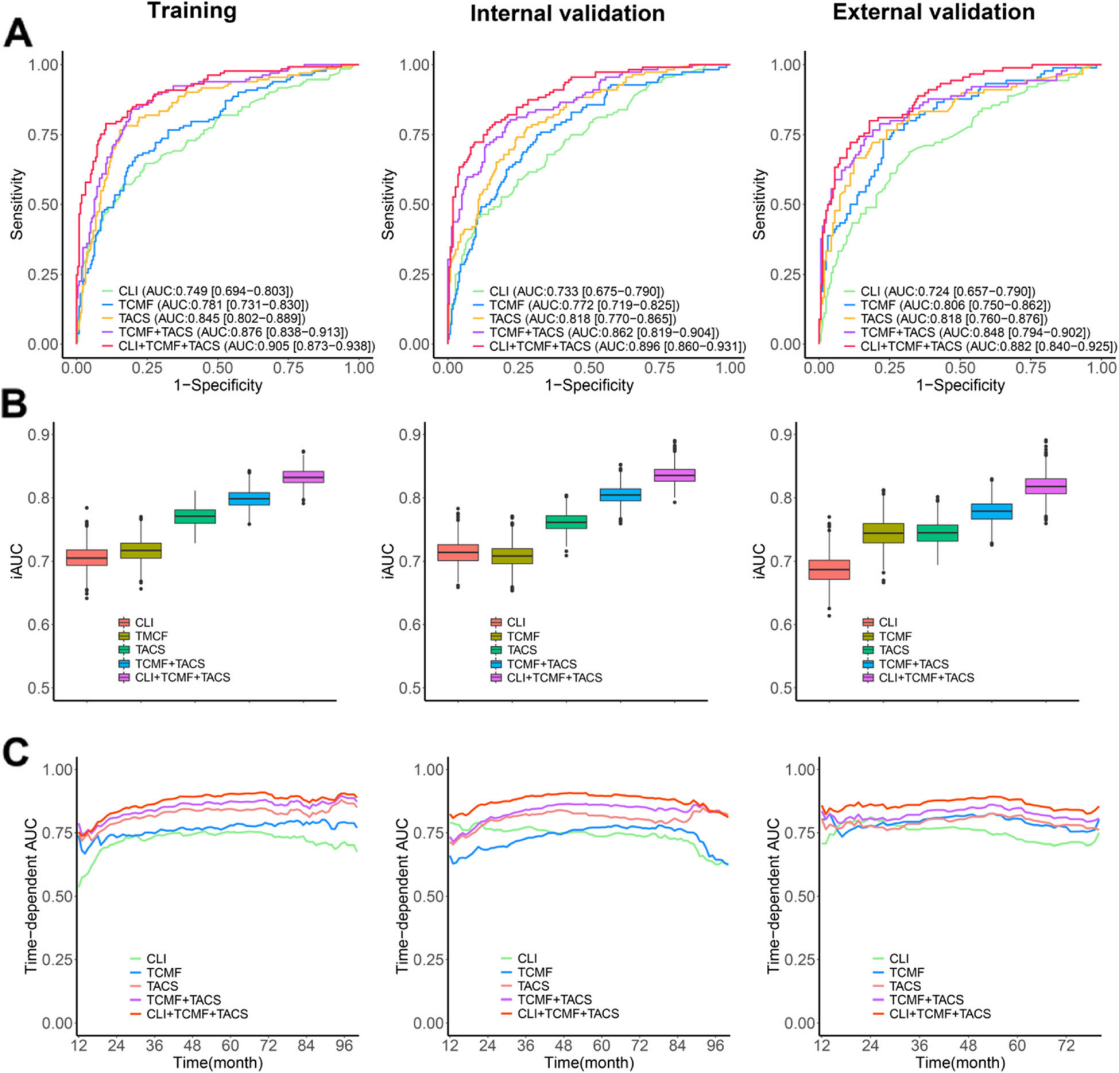
**A** ROC curves of the CLI, TCMF, TACS, TCFM+TACS, and CLI+TCMF+TACS models predicting 5-year DFS in three cohorts. **B** Mean *iAUC* of the five prognostic models in the three cohorts. **C** Comparison of the five prognostic models by time-dependent area under ROC curves (*AUC*) in the three cohorts

Model performance was primarily evaluated using receiver operating characteristic (ROC) analysis in the training, internal validation, and external validation cohorts. The TCMF model (*AUC* at 5-year DFS, 0.781; *95% CI*, 0.731 to 0.831) performs better than the CLI model (0.749; *95% CI*, 0.694 to 0.803), but is inferior to the TACS model (0.845, *95% CI*, 0.802 to 0.889) in the training cohort (Fig. 3A). The input into the CLI model are clinical risk factors including age, molecular subtype, tumor size, nodal status, histological grade, clinical stage, chemotherapy, and radiation therapy, and learning procedure is based on Cox proportional hazard regression. We included all clinical parameters to show that TACS-score or TMCF-score has a good prediction performance. These models were also applied to the internal validation and external validation cohorts to examine their generalizability. We found that the prediction performance of these models is generally stable on the two validation cohorts.

**Fig. 4:**
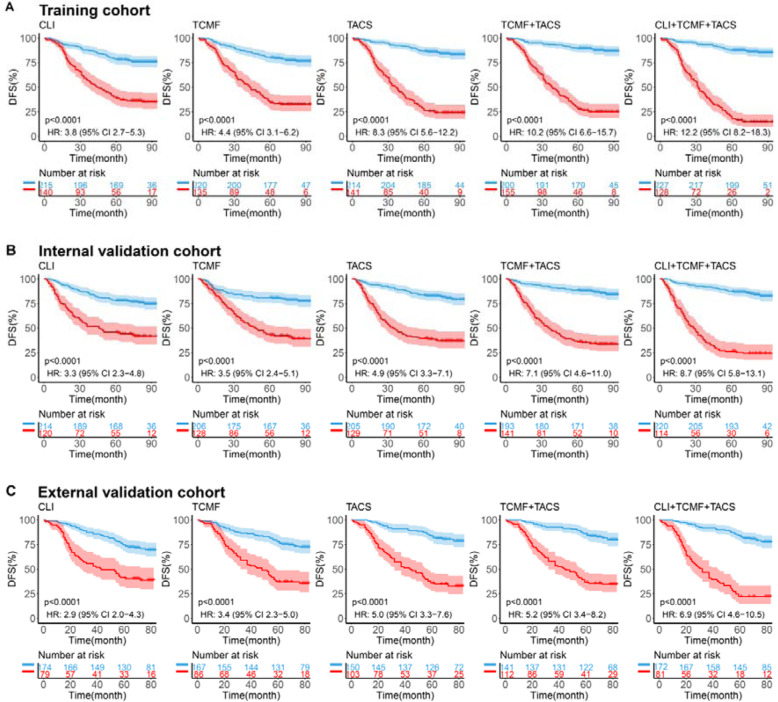
Kaplan-Meier curves according to the CLI model, TCMF model, TACS model, TCMF+TACS model, and CLI+TCMF+TACS model in three cohorts. The patients were divided into low-risk and high-risk groups using the optimal cutoff value of the five models. *P* values were calculated using the log-rank test. Blue lines show subjects with low risk, and red lines show subjects with high risk. **A** Training cohort (355 patients). **B** Internal validation cohort (334 patients). **C** External validation cohort (253 patients). DFS disease-free survival, HR hazard ratio

Moreover, patients were divided into low-risk and high-risk groups according to the cutoff value at the 5-year time point. The optimal cutoff value was determined by the maximum Youden index in the training cohort obtained by the ROC curve analysis. Survival differences between the low-risk and high-risk groups in each cohort were evaluated by Kaplan-Meier survival analysis. The predicted high-risk group has worse DFS than the low-risk group as shown in Fig. 4. As to the risk stratification, the hazard ratio (*HR*) value of the TCMF model is higher than that of the CLI model in the three cohorts, indicating that its risk stratification ability is superior to the clinical prognostic factors (*HR*, training 4.4 vs. 3.8, internal validation 3.5 vs. 3.3, external validation 3.4 vs. 2.9).

### Difference between TCMF and TACS models

In order to elucidate the predictive performance of these models among different subgroups, we conducted a number of subgroup analyses classified by the clinical variables. We evaluated the ability of risk stratification and prediction of 5-year DFS of different models in different subgroups (Table 2). The CLI model demonstrates poor predictive performance, and the performance was relatively consistent in each subgroup. The distribution of clinicopathological characteristics did not alter significantly between the high-risk and low-risk groups in the three cohorts. The TCMF model achieves better performance than the CLI model and significantly improves the classification accuracy, especially in some subgroups of low-risk variables (luminal A: *HR*, 5.738, *AUC*, 0.827; luminal B: *HR*, 4.647, *AUC*, 0.835; grade G1: *HR*, 5.206, *AUC*, 0.811; grade G2: *HR*, 4.122, *AUC*, 0.794; ER-positive: *HR*, 5.174, *AUC*, 0.833; PR-positive: *HR*, 6.026, *AUC*, 0.844; HER2-negative: *HR*, 4.083, *AUC*, 0.790). In contrast, as can be seen in Table 2, the TACS model appears to be particularly prominent in high-risk patients as discussed extensively in our previous publication [13]. When the two models (TCMF+TACS) are combined, they could complement each other and perform well in all patients, highlighting its general applicability (Additional file 1: Table S4, Fig. S3), and the full model (CLI+TCMF+TACS) achieves the best performance and further stratifies the low- and high-risk patients with outstanding values of *HR* (Additional file 1: Table S5).

**Table 2:** Comparison of 5-year prognostic performance by the five models for different clinicopathological subgroups

Variable	CLI		TCMF-score		TACS-score		TCMF+TACS		CLI+TCMF+TACS	
	***HR***	***AUC***	***HR***	***AUC***	***HR***	***AUC***	***HR***	***AUC***	***HR***	***AUC***
**Age**										
≤50	3.320	0.729	4.112	0.778	5.869	0.823	7.201	0.854	9.069	0.889
>50	3.282	0.739	3.323	0.788	5.999	0.833	7.653	0.872	9.243	0.902
**Molecular subtype**										
Luminal A	1.749	0.601	5.738	0.827	3.594	0.761	4.060	0.816	4.581	0.821
Luminal B	2.789	0.720	4.647	0.835	6.042	0.838	7.490	0.889	9.296	0.901
HER2-enriched	4.386	0.769	2.541	0.719	9.151	0.862	10.000	0.868	11.716	0.917
Triple negative	3.431	0.735	2.404	0.696	6.285	0.863	10.009	0.859	9.107	0.893
**Tumor size**										
≤2cm	2.824	0.683	3.959	0.782	5.976	0.818	7.001	0.855	6.899	0.872
2–5cm	3.150	0.725	3.987	0.789	6.209	0.839	8.264	0.871	10.631	0.897
>5cm	23.874	0.859	2.314	0.818	3.964	0.844	4.425	0.890	8.767	0.942
**Nodal status**										
0	2.874	0.628	3.343	0.751	5.443	0.820	6.808	0.856	8.250	0.880
1–3	1.681	0.593	4.330	0.799	3.871	0.758	5.216	0.813	6.086	0.829
≥4	4.332	0.728	2.826	0.788	6.337	0.865	7.433	0.881	10.753	0.910
**Clinical stage**										
I	4.024	0.615	3.299	0.741	4.071	0.797	5.042	0.833	6.546	0.865
II	1.881	0.609	4.143	0.790	5.256	0.798	7.012	0.844	8.048	0.862
III	3.773	0.724	2.722	0.775	6.102	0.863	7.125	0.876	9.500	0.904
**Histological grade**										
G1	3.214	0.703	5.206	0.811	4.889	0.769	8.407	0.849	10.520	0.876
G2	3.422	0.725	4.122	0.794	6.095	0.840	7.098	0.866	8.016	0.893
G3	2.810	0.733	2.639	0.749	6.528	0.845	8.775	0.874	10.677	0.898
**ER status**										
Positive	2.989	0.721	5.174	0.833	5.187	0.811	6.408	0.866	8.350	0.889
Negative	3.820	0.752	2.495	0.709	7.722	0.864	10.056	0.868	10.508	0.906
**PR status**										
Positive	3.116	0.721	6.026	0.844	5.178	0.807	7.354	0.865	8.698	0.891
Negative	3.416	0.744	2.473	0.719	7.219	0.859	8.012	0.867	9.562	0.899
**HER2 status**										
Negative	3.435	0.736	4.083	0.790	5.158	0.818	7.250	0.857	8.952	0.890
Positive	2.993	0.726	3.209	0.776	8.230	0.852	8.506	0.881	9.530	0.903

Note: *ER*, estrogen receptor; *PR*, progesterone receptor

To further examine whether there was a specific benefit of these models, we stratified the patients into various subgroups (ER-positive group: luminal, ER+/N−, ER+/HER2−, ER+/N−/HER2−, ER+/PR+; ER-negative group: non-luminal, ER−/N+, ER−/HER2+, ER−/N+/HER2+, ER−/PR−). The performance of the CLI model is mediocre in each subgroup (Additional file 1: Table S6). Surprisingly, for both risk stratification capability (*HR*) and prediction accuracy (*AUC*), the TCMF model is particularly outstanding in the ER-positive and lower-risk combination subgroup, and the TACS model is counter-matched in the ER-negative and higher-risk combination subgroup (Additional file 1: Table S6). Similarly, the TCMF+TACS model is quite robust in all ER-positive and ER-negative subgroups (*HR*, *AUC* in the Additional file 1: Table S6, C-index in the Additional file 1: Fig. S4). The differential benefit of the TCMF/TACS model is also revealed by the increases in *HR*, *AUC* from the CLI model to the full model (Additional file 1: Table S6).

### Combination prediction of TCMF- and TACS-score

When TCMF is combined with TACS, the *AUC* increases to 0.876 (*95% CI*, 0.838 to 0.913) (Fig. 3A). The accuracy, sensitivity, and specificity of TCMF+TACS are also universally better than other single models. The full model achieves more improved *AUC*, accuracy, and specificity, but reduces sensitivity, which may be due to the low sensitivity of the CLI model (Table 3). High specificity would help the model avoid misidentifying low-risk patients as high risk, i.e., few low-risk individuals are diagnosed as a high-risk group, while good sensitivity allows the model to not miss high-risk patients, i.e., most high-risk patients are correctly diagnosed as a high-risk group.

**Table 3:** The performance comparison of different models for predicting 5-year disease-free survival

Model	Training cohort				Internal validation cohort				External validation cohort			
	***AUC***	***ACC***	***SEN***	***SPE***	***AUC***	***ACC***	***SEN***	***SPE***	***AUC***	***ACC***	***SEN***	***SPE***
CLI	74.9	71.5	64.7	75.7	73.3	69.5	58.0	75.2	72.4	69.6	51.1	79.8
TCMF-score	78.1	74.6	66.9	79.3	77.2	71.9	65.2	75.2	80.6	73.1	60.0	80.4
TACS-score	84.5	81.4	78.2	83.3	81.8	74.6	69.6	77.0	81.8	75.9	73.3	77.3
TCMF+TACS	87.6	82.0	84.2	80.6	86.2	78.1	80.4	77.0	84.8	76.3	78.9	74.9
CLI+TCMF+TACS	90.5	85.6	79.0	89.6	89.6	82.6	75.0	86.5	88.2	82.2	70.0	89.0

Note: *AUC*, area under the receiver operating characteristic curve; *ACC*, accuracy; *SEN*, sensitivity; *SPE*, specificity

**Fig. 5:**
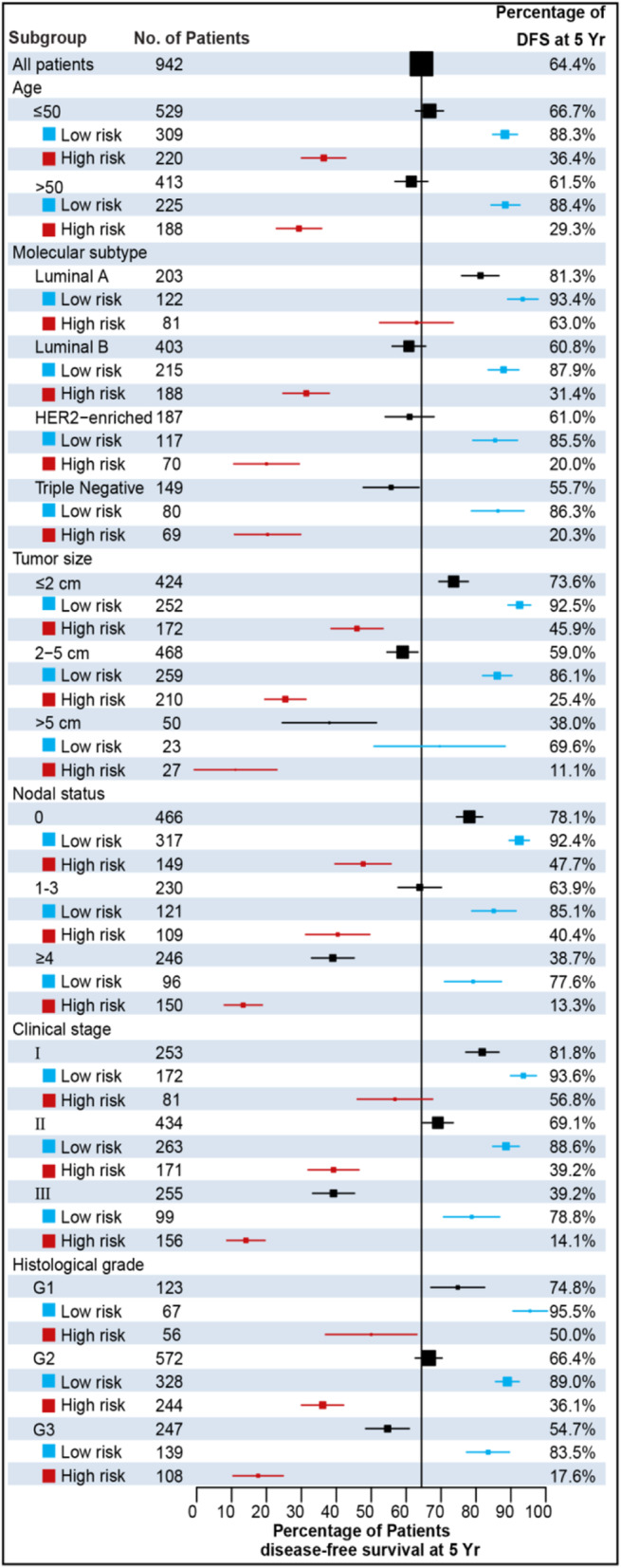
Forest plot shows the proportion of patients with disease-free survival at 5 years. For each subgroup, the low- and high-risk categories were obtained according to the optimal cutoff value of the TCMF+TACS-score. Patients with a low-risk score have less frequent recurrences at 5 years

The TCMF+TACS model predicts 5-year DFS for all categories by six clinical variables (Fig. 5). Patients with a low-risk score have less frequent recurrence at 5 years than these with a high-risk score. The personalized prognostic potential of the TCMF+TACS-score implies that we could triage all patients by the six clinical variables into low- and high-risk groups with diverged stratification of a 5-year DFS rate. Moreover, not all patients with small tumors (patients with a tumor 2 cm in diameter or smaller) and luminal A patients are at low risk. Those patients with a high-risk TCMF+TACS-score have a 54.1 and 37.0% risk of recurrence at 5 years, respectively.

To further evaluate the prognostic performance of different models, we calculated an integrated cumulative/dynamic area under the curve (*iAUC*) for the five models, respectively, being 70.6%, 71.7%, 77.1%, 79.8%, and 83.3% in the training cohort (Fig. 3B). Applying the models to the internal and external validation cohorts’ results in *iAUC* values of 71.4%, 70.9%, 75.9%, 80.5%, 83.6%, and 68.7%, 74.5%, 74.4%, 77.9%, 81.8%, respectively. Figure 3C shows the *AUC* for time-dependent ROC performance, and the performance of these models in predicting recurrence risk remains stable with only small fluctuation in time-dependent *AUC* values as we move away from the time point of diagnosis. These results highlight the importance of tumor-stroma spatial patterns.

As illustrated in Fig. 4A, the CLI model, TCMF model, TACS model, TCMF+TACS, and CLI+TCMF+TACS deliver an increasing *HR* from ~4 to ~12, revealing an increasing capacity for risk stratification. The TCMF+TACS model shows significantly better risk stratification than the CLI model (*HR* = 10.2 [6.6–15.7], *P* < 0.0001 vs. 3.8 [2.7–5.3], *P* < 0.0001), and the full model gains the highest *HR* (12.2 [8.2–18.3], *P* < 0.0001) and achieves the best performance in terms of *AUC* at 5-year DFS (Figs. 3 and 4A). To confirm that the TCMF+TACS model has an outstanding prognostic value in different populations, we further extended it to the internal and external validation cohorts and acquired the similar results (Figs. 3 and 4B, C). Thus, high discriminatory accuracy and super risk stratification ability validate TCMF+TACS-score as a strong DFS prognosticator.

Moreover, we propose that the combined analysis of CLI, TCMF, and TACS can improve the prognostic stratification of breast cancer patients by evaluating the high and low risks of different models. The risk groups of CLI^low^/TCMF^low^/TACS^low^ represent that primary breast cancer was controlled by standard treatment with improved DFS. In contrast, the risk groups of CLI^high^/TCMF^high^/TACS^high^ would represent a population of patients at risk for recurrence. Therefore, as can be seen from the Kaplan-Meier curves (Additional file 1: Fig. S5, upper left), low-risk groups of TCMF and TACS are associated with a higher DFS rate regardless of the CLI risk category. On the contrary, there is a poorer DFS rate when TCMF and TACS are both high (Additional file 1: Fig. S5, bottom right).

In addition, TCMF and TACS-score remain as independent prognostic factors by the multivariate analysis of DFS (Additional file 1: Table S1), along with the clinicopathologic biomarkers such as molecular subtype, tumor size, nodal status, and chemotherapy. Other clinical factors, such as age, clinical stage, histological grade, and radiation therapy, are excluded because they are not significantly associated with DFS in the univariate and multivariate Cox proportional hazard regression analyses (*P* > 0.05). Then, a clinically applicable nomogram integrating these independent prognostic factors was developed to predict 1-year, 3-year, and 5-year DFS in the training cohort (Additional file 1: Fig. S6A). Calibration plots (Additional file 1: Fig. S6B) show that nomogram performs well (C-index 0.88, 0.86–0.91 for the training cohort; 0.85, 0.82–0.89 for the internal validation cohort; and 0.85, 0.81–0.88 for the external validation cohort) and displays excellent agreement between the observed and predicted rates in the three cohorts (proximity to 45° diagonal line).

### Clinical applications

Finally, we used the CLI, TCMF, TACS, and CLI+TCMF+TACS models to evaluate the effect of postoperative adjuvant treatment on different recurrence risk groups stratified via the treatment guideline in China, which agrees with the 10th St Gallen expert consensus [35]. This guideline allowed 590 patients to be classified as minimum risk (*n* = 28) or moderate risk (*n* = 562) (defined together as low risk) for less aggressive treatment, and 352 patients as high risk for more aggressive treatment (Additional file 1: Table S7), and thereby may cause undertreatment up to 139 patients and overtreatment up to 156 patients, demonstrating a significant percentage of patients may receive inappropriate therapy. The CLI model would lower 1 undertreated patient and 14 overtreated patients. By contrast, the full model would reduce the undertreated patients by 56 and the overtreated patients by 85 (Additional file 1: Table S7). Therefore, there may be inadequate treatment or overtreatment for some patients after standard treatment.

On the other hand, we re-stratified the two group patients (low- and high-risk groups by clinical guideline) by the five models (CLI, TCMF, TACS, TCMF+TACS, and CLI+TCMF+TACS models). The full model (CLI+TCMF+TACS) presents higher hazard ratios (*HR*, 7.0 [5.1–9.7] in patients with low risk by clinical guideline, 9.6 [6.4–14.0] in patients with high risk by clinical guideline) (Additional file 1: Fig. S9). In patients with low/intermediate recurrence risk by clinical guideline, 79.7% of patients (*n* = 590) is still classified as low risk and 20.3% of patients (*n* = 590) is reclassified as high risk by the full model, and in patients with high recurrence risk by clinical guideline, 42.3% of patients (*n* = 352) is reclassified as low risk and 57.7% of patients (*n* = 352) is still classified as high risk. Thus, to some extent, the full model may complement the Chinese treatment guideline and improve the choice of personalized adjuvant therapy.

## Discussion

At present, conventional clinical management of breast cancer mainly depends on the traditional prognostic factors, including patient’s age, molecular subtype, tumor size, lymph node metastasis, clinical stage, and histological grade, in addition to estrogen receptor (ER), progesterone receptor (PR), and HER2 [36, 37]. These biomarkers are valuable for predicting local recurrence and distant metastasis [38]. However, patients with the same pathological features often have different outcomes, suggesting that the current clinical factors are inadequate for predicting prognosis [39]. Therefore, the search for new prognostic and predictive biomarkers is motivated to promote the prognostic evaluation and improve patient outcome. In our previous study, we found that TACS1-8 emerge as a tumor microenvironment-based structural prognosticator, but these large-scale collagen patterns are macroscopic morphologies observed from multiphoton images [13].

High-throughput collagen microscopic features including morphological and textural features can be extracted through image processing to quantify the differences between tissues. The morphological features could quantify shape-related properties, such as collagen area, number, length, width, straightness, crosslink density, crosslink space, and orientation. Recently, Sprague et al. found that multiple morphological features of collagen fibers around DCIS were associated with DCIS recurrence risk [40]: patients with greater collagen width and density had a lower risk of recurrence, while patients with higher fiber straightness and distance to the nearest two fibers had a higher risk of recurrence. However, Case et al. reported that collagen width is associated with poor outcome by multivariate analysis [41]. The texture features, including histogram, gray-level co-occurrence matrix (GLCM), and Gabor wavelet transformation feature, have been proved to be popular features in biomedical image analysis [19, 22, 42–44]. Texture analysis, which is based on first-order and second-order statistics, can be used to extract the image features associated with the structural and biochemical changes in collagen networks and quantitatively track the alterations correlated with collagen remodeling [45]. GLCM is a second-order statistical texture feature that reflects the spatial heterogeneity of collagen fibers with five different displacements of pixels and four different directions, and Gabor wavelet transformation is also a type of textural analysis that could reflect the spatial relationship of images in different scales and orientations after the convolution of images. Texture analysis has been employed extensively in magnetic resonance imaging (MRI) for distinguishing between breast cancer subtypes [46], differentiating between benign and malignant lesions [47], and evaluating the relationship between textural features and survival outcomes of patients with breast cancer [48], but not for evaluating the collagen spatial structure because of the limitation in resolution of MRI [49]. In this study, morphological and textural analyses were used for extracting the microscopic features of collagen fibers in high spatial resolution MPM images.

We used computer-assisted image feature processing to automatically extract the corresponding microscopic feature of each TACS (TCMF), and then made use of the least absolute shrinkage and selection operator (LASSO) regression to choose the most robust features to acquire a TCMF-score. Statistical analyses show that TCMF-score is a strong independent prognostic indicator for predicting DFS. Interestingly, we found different prognostic values for subgroups when the TACS and TCMF were used for evaluating the accuracy (*AUC*) of diagnosing 5-year DFS and risk stratification ability. TACS-score would be helpful to recognize a significant percentage of high-risk patients classified through the clinicopathological factors. By contrast, TCMF-score performs better for the low-risk patients, especially for hormone-positive breast cancer, which may compete with the popular multigene assays on prognosis [50, 51]. As we all know, microstructural changes will precede the changes of macrostructure. The changes in microstructure may occur in the early stage of diseases, but cannot be observed visually. It is these microstructural changes, rather than the macrostructural changes, that may have the sensitivity to signify important features for detecting early lesions [52]. This may be the reason why the microscopic collagen features are more suitable for identifying low-risk patients, while the macroscopic collagen patterns are more suitable for identifying high-risk patients. It is a promising method for the early detection of lesions by microstructural changes. What is more, the TCMF+TACS-score is far better than the well-established clinicopathological factors in prognostic performance, highlighting the obbligato role of the tumor microenvironment in cancer progression. This study would extend our previous research on TACS and highlight the importance of microscopic collagen morphology changes.

## Conclusions

In summary, we used computer-assisted image processing to automatically extract the corresponding microscopic features of each TACS (TCMF) and then utilized the LASSO regression to screen out the most robust features to establish a TCMF-score. TCMF and TACS would reflect the microscopic and macroscopic morphology changes of collagen fibers in the breast tumor microenvironment. A combination of TCMF-score with TACS-score could be used for predicting individual disease-free survival rate with good statistical significance, high discriminatory accuracy, and superior risk stratification ability. With the increasing automation of image processing, TCMF+TACS screening has great potential to become a clinical diagnostic tool, providing more accurate prognosis information.

## Supplementary Information

**Additional file 1: Supplementary Methods:** 1.1 Multiphoton imaging system. 1.2 TACS-score calculation formula. 1.3 Morphological features. 1.4 Texture features. 1.5 TCMF-score calculation formula. **Figures S1-S10: Fig. S1.** Recruitment pathways for patients. **Fig. S2**. Distribution histogram of TCMF-score, TACS-score and TCMF+TACS-score. **Fig. S3**. Hazard ratio of 5-year recurrence for all patients according to the TCMF+TACS-score classifier in different subgroups stratified by clinical parameters. **Fig. S4**. Forest plot shows the concordance index (C-index) of different prediction models (TCMF, TACS, and TCMF+TACS) in the different risk combination patients. **Fig. S5**. Kaplan-Meier survival curves for comparing the risk stratification ability of the CLI-score under four different situations (TACS-low risk and TCMF-low risk, TACS-high risk and TCMF-low risk, TACS-low risk and TCMF-high risk, TACS-high risk and TCMF-high risk). **Fig. S6**. Nomogram and Calibration curves. **Fig. S7**. TCMF selection using LASSO cox regression analysis. **Fig. S8**. Schematic of extracting TACS corresponding microscopy features (TCMF). **Fig. S9**. Kaplan-Meier curves of DFS according to the CLI, TCMF, TACS, TCMF+TACS, and CLI+TCMF+TACS models for the low risk and high risk patients classified by the treatment guideline. **Fig. S10**. Representative MPM images of three patients to illustrate the extraction of TCMF. **Tables S1-S11: Table S1**. Univariate and multivariate Cox proportional hazards regression analyses of the association of variables with DFS in the training cohort. **Table S2**. Univariate and multivariate Cox proportional hazards regression analyses of the association of variables with DFS in the internal validation cohort. **Table S3**. Univariate and multivariate Cox proportional hazards regression analyses of the association of variables with DFS in the external validation cohort. **Table S4**. Risk stratification ability of TCMF+TACS-score in subgroups of the three cohorts. **Table S5**. Risk stratification ability of the five prediction models in three cohorts. **Table S6**. Prognosis performance of the five models at 5-year DFS in patients stratified by the combination of different risk factors. **Table S7**. The number of likely undertreated (orange-highlighted) and overtreated (purple-highlighted) patients according to the Chinese treatment guideline and four models. **Table S8**. Morphological features. **Table S9**. First order intensity histogram features. **Table S10**. Grey-level co-occurrence matrix-based features. **Table S11**. Gabor wavelet transform features.

**Additional file 2.** TCMF1-142, TCMF-score.

**Additional file 3.** TACS1-8, TACS-score.

**Additional file 4.** Clinical Factors, CLI-score.

## Data Availability

All data generated or analyzed during this study are included in this article and its supplementary information files (Additional file 2, Additional file 3, and Additional file 4). The code to extract the TCMFs is available from the corresponding authors on reasonable request.

## Abbreviations

*AUC*: Area under the curve
CLI: Clinical
DCIS: Ductal carcinoma in situ
DFS: Disease-free survival
ECM: Extracellular matrix
ER: Estrogen receptor
FFPE: Formalin-fixed paraffin-embedded
GLCM: Gray-level co-occurrence matrix
H&E: Hematoxylin and eosin
HR: Hazard ratio
LASSO: Least absolute shrinkage and selection operator
MPM: Multiphoton microscopy
MRI: Magnetic resonance imaging
PR: Progesterone receptor
ROC: Receiver operating characteristic
ROI: Region of interest
SHG: Second harmonic generation
TACS: Tumor-associated collagen signatures
TCMF: TACS corresponding microscopic feature
TPEF: Two-photon excited fluorescence

## References

[1] Chaffer CL, Weinberg RA (2011). A perspective on cancer cell metastasis. Science..

[2] Kakkad SM, Solaiyappan M, Argani P, Sukumar S, Jacobs LK, Leibfritz D, Bhujwalla Z, Glunde K (2012). Collagen I fiber density increases in lymph node positive breast cancers: pilot study. J Biomed Opt.

[3] Frantz C, Stewart KM, Weaver VM (2010). The extracellular matrix at a glance. J Cell Sci.

[4] Charras G, Sahai E (2014). Physical influences of the extracellular environment on cell migration. Nat Rev Mol Cell Biol.

[5] Lochter A, Bissell MJ (1995). Involvement of extracellular matrix constituents in breast cancer. Semin Cancer Biol.

[6] Grossman M, Ben-Chetrit N, Zhuravlev A, Afik R, Bassat E, Solomonov I, Yarden Y, Sagi I (2016). Tumor cell invasion can be blocked by modulators of collagen fibril alignment that control assembly of the extracellular matrix. Cancer Res.

[7] Azimzade Y, Saberi AA, Sahimi M (2019). Regulation of migration of chemotactic tumor cells by the spatial distribution of collagen fiber orientation. Phys Rev E.

[8] Tamimi SO, Ahmed A (1987). Stromal changes in invasive breast carcinoma: an ultrastructural study. J Pathol.

[9] Radisky D, Muschler J, Bissell MJ (2002). Order and disorder: the role of extracellular matrix in epithelial cancer. Cancer Investig.

[10] Provenzano PP, Eliceiri KW, Campbell JM, Inman DR, White JG, Keely PJ (2006). Collagen reorganization at the tumor-stromal interface facilitates local invasion. BMC Med.

[11] Han W, Chen S, Yuan W, Fan Q, Tian J, Wang X, Chen L, Zhang X, Wei W, Liu R, Qu J, Jiao Y, Austin RH, Liu L (2016). Oriented collagen fibers direct tumor cell intravasation. Proc Natl Acad Sci USA.

[12] Conklin MW, Eickhoff JC, Riching KM, Pehlke CA, Eliceiri KW, Provenzano PP, Friedl A, Keely PJ (2011). Aligned collagen is a prognostic signature for survival in human breast carcinoma. Am J Pathol.

[13] Xi G, Guo W, Kang D, Ma J, Fu F, Qiu L, Zheng L, He J, Fang N, Chen J, Li J, Zhuo S, Liao X, Tu H, Li L, Zhang Q, Wang C, Boppart SA, Chen J (2021). Large-scale tumor-associated collagen signatures identify high-risk breast cancer patients. Theranostics..

[14] Altendorf H, Decencière E, Jeulin D, Peixoto PDS, Deniset-Besseau A, Angelini E, Mosser G, Schanne-Klein MC (2012). Imaging and 3D morphological analysis of collagen fibrils. J Microsc.

[15] Bredfeldt JS, Liu Y, Pehlke CA, Conklin MW, Eliceiri KW (2014). Computational segmentation of collagen fibers from second-harmonic generation images of breast cancer. J Biomed Opt.

[16] Falzon G, Pearson S, Murison R (2008). Analysis of collagen fibre shape changes in breast cancer. Phys Med Biol.

[17] Hu W, Li H, Wang C, Gou S, Fu L (2012). Characterization of collagen fibers by means of texture analysis of second harmonic generation images using orientation-dependent gray level co-occurrence matrix method. J Biomed Opt.

[18] Bredfeldt JS, Liu Y, Conklin MW, Keely PJ, Mackie TR, Eliceiri KW (2014). Automated quantification of aligned collagen for human breast carcinoma prognosis. J Pathol Inform.

[19] Chen D, Chen G, Jiang W, Fu M, Liu W, Sui J, Xu S, Liu Z, Zheng X, Chi L, Lin D, Li K, Chen W, Zuo N, Lu J, Chen J, Li G, Zhuo S, Yan J (2019). Association of the collagen signature in the tumor microenvironment with lymph node metastasis in early gastric cancer. JAMA Surg.

[20] Dempster AP, Laird NM, Rubin DB (1977). Maximum likelihood from incomplete data via the EM algorithm. J Roy Stat Soc B.

[21] Stein AM, Vader DA, Jawerth LM, Weitz DA, Sander LM (2008). An algorithm for extracting the network geometry of three-dimensional collagen gels. J Microsc.

[22] Xu S, Kang CH, Gou X, Peng Q, Yan J, Zhuo S, Cheng CL, He Y, Kang Y, Xia W (2016). Quantification of liver fibrosis via second harmonic imaging of the Glisson’s capsule from liver surface. J Biophotonics.

[23] Frisch KE, Duenwald-Kuehl SE, Kobayashi H, Chamberlain CS, Lakes RS, Vanderby JR (2012). Quantification of collagen organization using fractal dimensions and Fourier transforms. Acta Histochem.

[24] Haralick RM, Shanmugam K (1973). Textural features for image classification. IEEE Trans Syst Man Cybern.

[25] Daugman JG (1988). Complete discrete 2-D Gabor transforms by neural networks for image analysis and compression. IEEE Trans Acoust Speech Signal Process.

[26] Grigorescu SE, Petkov N, Kruizinga P (2002). Comparison of texture features based on Gabor filters. Ieee T Image Process.

[27] Chauhan A, Chauhan D, Rout C (2014). Role of gist and PHOG features in computer-aided diagnosis of tuberculosis without segmentation. PLoS ONE.

[28] Shahraki HR, Salehi A, Zare N (2015). Survival prognostic factors of male breast cancer in Southern Iran: a LASSO-Cox regression approach. Asian Pac J Cancer Prev.

[29] Tibshirani R (1996). Regression shrinkage and selection via the lasso. J R Statist Soc B.

[30] Tibshirani R. The lasso method for variable selection in the Cox model. Stat Med. 1997;16(4):385–95. 10.1002/(SICI)1097-0258(19970228)16:4<385::AID-SIM380>3.0.CO;2-3. 10.1002/(sici)1097-0258(19970228)16:4<385::aid-sim380>3.0.co;2-3 9044528

[31] Simon N, Friedman J, Hastie T, Tibshirani R (2011). Regularization paths for Cox’s proportional hazards model via coordinate descent. J Stat Softw.

[32] Zhu Z, Li L, Ye Z, Fu T, Du Y, Shi A, Wu D, Li K, Zhu Y, Wang C (2017). Prognostic value of routine laboratory variables in prediction of breast cancer recurrence. Sci Rep.

[33] Graesslin O, Abdulkarim BS, Coutant C, Huguet F, Gabos Z, Hsu L, Marpeau O, Uzan S, Pusztai L, Strom EA, Hortobagyi GN, Rouzier R, Ibrahim NK (2010). Nomogram to predict subsequent brain metastasis in patients with metastatic breast cancer. J Clin Oncol.

[34] Kim Y, Margonis GA, Prescott JD, Tran TB, Postlewait LM, Maithel SK, Wang TS, Evans DB, Hatzaras I, Shenoy R, Phay JE, Keplinger K, Fields RC, Jin LX, Weber SM, Salem AI, Sicklick JK, Gad S, Yopp AC, Mansour JC, Duh QY, Seiser N, Solorzano CC, Kiernan CM, Votanopoulos KI, Levine EA, Poultsides GA, Pawlik TM (2016). Nomograms to predict recurrence-free and overall survival after curative resection of adrenocortical carcinoma. JAMA Surg.

[35] Goldhirsch A, Wood W, Gelber R, Coates A, Thürlimann B, Senn H-J, Members P (2007). Progress and promise: highlights of the international expert consensus on the primary therapy of early breast cancer 2007. Ann Oncol.

[36] Mori I, Yang Q, Kakudo K (2002). Predictive and prognostic markers for invasive breast cancer. Pathol Int.

[37] Hayes DF, Isaacs C, Stearns V (2001). Prognostic factors in breast cancer: current and new predictors of metastasis. J Mammary Gland Biol.

[38] Rakha EA, El-Sayed ME, Green AR, Lee AH, Robertson JF, Ellis IO (2007). Prognostic markers in triple-negative breast cancer. Cancer..

[39] Dowsett M, Houghton J, Iden C, Salter J, Farndon J, A'hern R, Sainsbury R, Baum M (2006). Benefit from adjuvant tamoxifen therapy in primary breast cancer patients according oestrogen receptor, progesterone receptor, EGF receptor and HER2 status. Ann Oncol.

[40] Sprague BL, Vacek PM, Mulrow SE, Evans MF, Trentham-Dietz A, Herschorn SD, James TA, Surachaicharn N, Keikhosravi A, Eliceiri KW (2021). Collagen organization in relation to ductal carcinoma in situ pathology and outcomes. Cancer Epidemiol Prev Biomark.

[41] Case A, Brisson BK, Durham AC, Rosen S, Monslow J, Buza E, Salah P, Gillem J, Ruthel G, Veluvolu S (2017). Identification of prognostic collagen signatures and potential therapeutic stromal targets in canine mammary gland carcinoma. PLoS ONE.

[42] Chen D, Liu Z, Liu W, Fu M, Jiang W, Xu S, Wang G, Chen F, Lu J, Chen H, Dong X, Li G, Chen G, Zhuo S, Yan J (2021). Predicting postoperative peritoneal metastasis in gastric cancer with serosal invasion using a collagen nomogram. Nat Commun.

[43] Mostaço-Guidolin LB, Osei ET, Ullah J, Hajimohammadi S, Fouadi M, Li X, Li V, Shaheen F, Yang CX, Chu F (2019). Defective fibrillar collagen organization by fibroblasts contributes to airway remodeling in asthma. Am J Resp Crit Care.

[44] Hristu R, Eftimie LG, Stanciu SG, Tranca DE, Paun B, Sajin M, Stanciu GA (2018). Quantitative second harmonic generation microscopy for the structural characterization of capsular collagen in thyroid neoplasms. Biomed Opt Express.

[45] Mostaço-Guidolin LB, Ko AC-T, Wang F, Xiang B, Hewko M, Tian G, Major A, Shiomi M, Sowa MG (2013). Collagen morphology and texture analysis: from statistics to classification. Sci Rep.

[46] Waugh SA, Purdie CA, Jordan LB, Vinnicombe S, Lerski RA, Martin P, Thompson AM (2016). Magnetic resonance imaging texture analysis classification of primary breast cancer. Eur Radiol.

[47] Chen W, Giger ML, Li H, Bick U, Newstead GM (2010). Volumetric texture analysis of breast lesions on contrast-enhanced magnetic resonance images. Magn Reson Med.

[48] Kim JH, Ko ES, Lim Y, Lee KS, Han BK, Ko EY, Hahn SY, Nam SJ (2016). Breast cancer heterogeneity: MR imaging texture analysis and survival outcomes. Radiology..

[49] Ross KA, Williams RM, Schnabel LV, Mohammed HO, Potter HG, Bradica G, Castiglione E, Pownder SL, Satchell PW, Saska RA, Fortier LA (2013). Comparison of three methods to quantify repair cartilage collagen orientation. Cartilage..

[50] Goldstein LJ, Gray R, Badve S, Childs BH, Yoshizawa C, Rowley S, Shak S, Baehner FL, Ravdin PM, Davidson NE, Sledge GW, Perez EA, Shulman LN, Martino S, Sparano JA (2008). Prognostic utility of the 21-gene assay in hormone receptor–positive operable breast cancer compared with classical clinicopathologic features. J Clin Oncol.

[51] Ring BZ, Seitz RS, Beck R, Shasteen WJ, Tarr SM, Cheang MC, Yoder BJ, Budd GT, Nielsen TO, Hicks DG (2006). Novel prognostic immunohistochemical biomarker panel for estrogen receptor–positive breast cancer. J Clin Oncol.

[52] Lee W, Moghaddam AO, Shen S, Phillips H, McFarlin B, Johnson AW, Toussaint K (2021). An optomechanogram for assessment of the structural and mechanical properties of tissues. Sci Rep.

