# Supplementary material for: Computer-assisted quantification of tumor-associated collagen signatures to improve the prognosis prediction of breast cancer
Source: BMC Med. 2021 Nov 18;19:273. doi: 10.1186/s12916-021-02146-7 (PMC8600902; doi:10.1186/s12916-021-02146-7)
Supplement: Supplementary file 1 — Additional file 1: Supplementary Methods: 1.1 Multiphoton imaging system. 1.2 TACS-score calculation formula. 1.3 Morphological features. 1.4 Texture features. 1.5 TCMF-score calculation formula. Figures S1-S10: Fig. S1. Recruitment pathways for patients. Fig. S2. Distribution histogram of TCMF-score, TACS-score and TCMF+TACS-score. Fig. S3. Hazard ratio of 5-year recurrence for all patients according to the TCMF+TACS-score classifier in different subgroups stratified by clinical parameters. Fig. S4. Forest plot shows the concordance index (C-index) of different prediction models (TCMF, TACS, and TCMF+TACS) in the different risk combination patients. Fig. S5. Kaplan-Meier survival curves for comparing the risk stratification ability of the CLI-score under four different situations (TACS-low risk and TCMF-low risk, TACS-high risk and TCMF-low risk, TACS-low risk and TCMF-high risk, TACS-high risk and TCMF-high risk). Fig. S6. Nomogram and Calibration curves. Fig. S7. TCMF selection using LASSO cox regression analysis. Fig. S8. Schematic of extracting TACS corresponding microscopy features (TCMF). Fig. S9. Kaplan-Meier curves of DFS according to the CLI, TCMF, TACS, TCMF+TACS, and CLI+TCMF+TACS models for the low risk and high risk patients classified by the treatment guideline. Fig. S10. Representative MPM images of three patients to illustrate the extraction of TCMF. Tables S1-S11: Table S1. Univariate and multivariate Cox proportional hazards regression analyses of the association of variables with DFS in the training cohort. Table S2. Univariate and multivariate Cox proportional hazards regression analyses of the association of variables with DFS in the internal validation cohort. Table S3. Univariate and multivariate Cox proportional hazards regression analyses of the association of variables with DFS in the external validation cohort. Table S4. Risk stratification ability of TCMF+TACS-score in subgroups of the three cohorts. Table S5. Risk stratification ability [file 12916_2021_2146_MOESM1_ESM.docx]

**Additional File 1**

**Computer-assisted quantification of tumor-associated collagen signatures to improve the prognosis** **prediction of breast cancer**

**Supplementary methods**

**1.1 Multiphoton imaging system**

The multiphoton microscopic imaging system used in this work has been described previously [13]. In brief, an upright microscope (LSM 880, Zeiss, Germany) equipped with a mode-locked femtosecond Ti: sapphire laser (Chameleon Ultra, Coherent) was used to obtain high-resolution images. In this study, 810nm linearly polarized light was selected for nonlinear optical imaging, and a Plan-Apochromat ×20 objective (NA = 0.8, Zeiss, Germany) was used for focusing the excitation beam into samples. A 32-channel GaAsP photomultiplier tube array detector was used for collecting second harmonic generation (SHG) signal (green color) in the wavelength range of 395 to 415 nm, and for the collection of two-photon excitation fluorescence (TPEF) signal (red color) in the wavelength range from 428 to 695 nm. To obtain a large-scale image, a fine focusing stage is used to translate the samples, and each large-scale image is stitched together from a series of x-y scan images. Each x-y scan image contains 512 × 512 pixels with a data depth of 12 bits. The lateral resolution is ~0.8 µm while the imaging field of view is about 0.5mm×0.5mm.

**1.2 TACS-score calculation formula**

We used ridge regression with cross validation to retrieve the coefficient of the TACS1-8, and then TACS-score was calculated for each patient using the linear combination of TACS percentages weighted by their regression coefficients.

TACS-score = (-1.10820 * TACS1) + (0.86111 * TACS2) – (2.20172 * TACS3) – (1.28742 * TACS4) + (0.65506 * TACS5) + (1.70003 * TACS6) + (0.30147 * TACS7) + (1.34240 * TACS8)

**1.3 Morphological features**

Eight morphological features were extracted, namely, the collagen percentage area, fiber density, average fiber length, average fiber width, average fiber straightness, crosslink density, average crosslink space and orientation (Table S8) [20-23]. In terms of morphological features, the segmentation algorithm based on Gaussian mixture model [20] was used to segment the SHG image into collagen fibers and background. Then, the binary mask image of the collagen fibers was processed by a well-established fiber network extraction algorithm [21] to track each fiber. After fiber extraction, the skeleton of each fiber were identified and represented by a list of ordered vertices [22]. If any vertex in the list belongs to more than one fiber, it would be recognized as a crosslink point. The vertex lists were used to calculate fiber density, length, width, straightness, crosslink density, crosslink space as summarized in Table S8. In addition, the orientation index of collagen alignment is quantified based on Fourier transform [23].

**1.4 Texture features**

Intensity based features, which are defined using first order statistics of the SHG pixel intensity histogram, consist of the mean, variance, skewness, kurtosis, energy and entropy (Table S9). We also include 80 gray-level co-occurrence matrix (GLCM)-based texture features [24] and 48 Gabor wavelet transform features [25]. The contrast, correlation, energy and homogeneity are calculated from the GLCM with five different displacements of pixels and four different directions at 0, 45, 90 and 135 degrees. The GLCM is a second-order statistical texture feature that is defined as matrix *P*(*i*, *j* | *d*, *θ*) to indicate the relative frequency with the intensity values of two pixels (*i* and *j*) in different spatial distance *d* and direction *θ*. *P*(𝑖, 𝑗) is the co-occurrence matrix by *d* and *θ*, 𝑥, 𝑦 denote the spatial coordinates of the pixel. *N_g_* refers to the number of discrete intensity levels in the image, 𝜇_x_(𝑖) is the mean of 𝑃_𝑥_(𝑖), 𝜇_y_(𝑗) is the mean of 𝑃_𝑦_(𝑗). σ_x_(𝑖) is the standard deviation of 𝑃_𝑥_(𝑖), and σ_y_(𝑗) is the standard deviation of 𝑃_y_(𝑗) (Table S10). To calculate the Gabor wavelet transform features (Table S11), we convolve the SHG image with Gabor filters at four scales and six different orientations at 0, 30, 60, 90, 120, and 150 degrees, and the mean and variation of the magnitude of the convolution over the image at each setting are calculated. Gabor wavelet transformation is a textural analysis that reflects spatial relationship of different scales and orientations after convolution of images [26]. The formula of two-dimensional Gabor function *g* (*x*, *y*) and its Fourier transform *G* (*u*, *v*) are as follows [27]:

$g \left( x,y \right)=\frac{1}{2\pi\sigma_{x}\sigma_{y}}exp\left[ -\frac{1}{2}\left( \frac{x^{2}}{\sigma_{x^{2}}}+\frac{y^{2}}{\sigma_{y^{2}}} \right)+2\pi jWx \right]$ (1)

$G\left( u,v \right)=exp\left\{ -\frac{1}{2}\left[ \frac{(u-W^{2})}{\sigma_{u^{2}}}+\frac{v^{2}}{\sigma_{v^{2}}} \right] \right\}$ (2)

Where *W* is the frequency of the Gabor function, $\sigma_{u}=\frac{1}{2}\pi\sigma_{x,} \sigma_{v}=\frac{1}{2}\pi\sigma_{y}$.

**1.5 TCMF-score calculation formula**

Due to the high dimension features, the variable selection is performed with least absolute shrinkage and selection operator (LASSO) Cox regression, which is a widely-used method for selection of the most useful prognostic markers for time-to-event analysis [28]. LASSO regression uses a L1 penalty to shrink some regression coefficient to exactly zero, with the degree of shrinkage dependent on a penalty parameter λ, called the tuning parameter. Ten-fold cross-validations are used to determine the optimal values of *λ*.

LASSO Cox regression coefficients are obtained through solving the equation:

$\hat{\beta}_{Lasso Cox}=arg max \left\{ PL \right.\left. \left( \beta\right)+\lambda\sum\left| \beta_{j} \right| \right\}$ (3)

Where $\hat{\beta}_{Lasso Cox}$ is the obtained parameters, $PL(\beta)$ is the Cox partial likelihood function, *λ* is positive constant value, and $\beta_{j}$ is the regression coefficient. Choosing the optimal *λ* is very important because the higher this value is, the more coefficients become zero, the model gets sparser, and also the higher the interpretability will be. In this study, the constant *λ* is set as 0.02038, with log (*λ*) = -3.893, and LASSO selects 14 nonzero coefficients. A formula is generated using a linear combination of selected features that are weighted by their respective LASSO coefficients $\hat{\beta}$, the formula is then used to calculate a score for each patient to reflect the risk of prognosis.

We applied LASSO algorithm jointly with the Cox survival model to implement a nested feature selection scheme based on the association between every TCMF feature and DFS. A formula is generated using a linear combination of the selected 14 features that are weighted by their respective LASSO coefficients and then is used to calculate TCMF-score for each patient.

TCMF-score = -0.30704* Area -0.15225*Number +0.10861*Orientation

+0.09697* Histogram variance -0.04821*Histogram entropy

+0.36665* GLCM_contrast_0°_3 pixel

+0.01091* GLCM_contrast_45°_5 pixel

+0.13102* GLCM_correlation_90°_4 pixel

+0.05917* GLCM_ correlation_135°_2 pixel

+0.08515* GLCM_ correlation_135°_4 pixel

-0.11233* Gabor_ variance_90°_2 scale

-0.20990* Gabor_ variance_120°_2 scale

-0.14594* Gabor_ variance_30°_3 scale

-0.00120* Gabor_ variance_120°_3 scale

**Supplementary Figures**


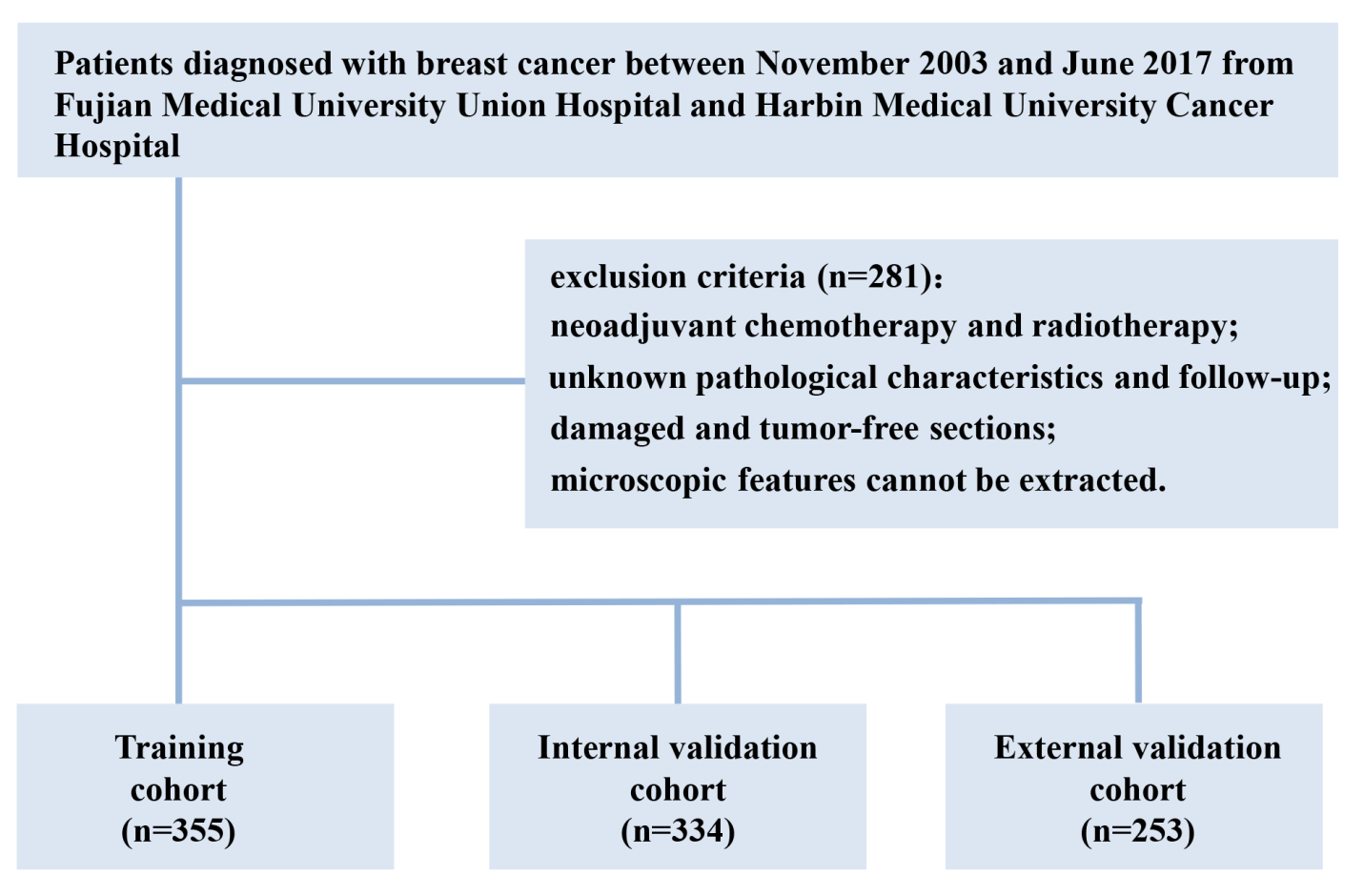


**Figure S1.** Recruitment pathways for patients in the training, internal validation, and external validation cohorts.


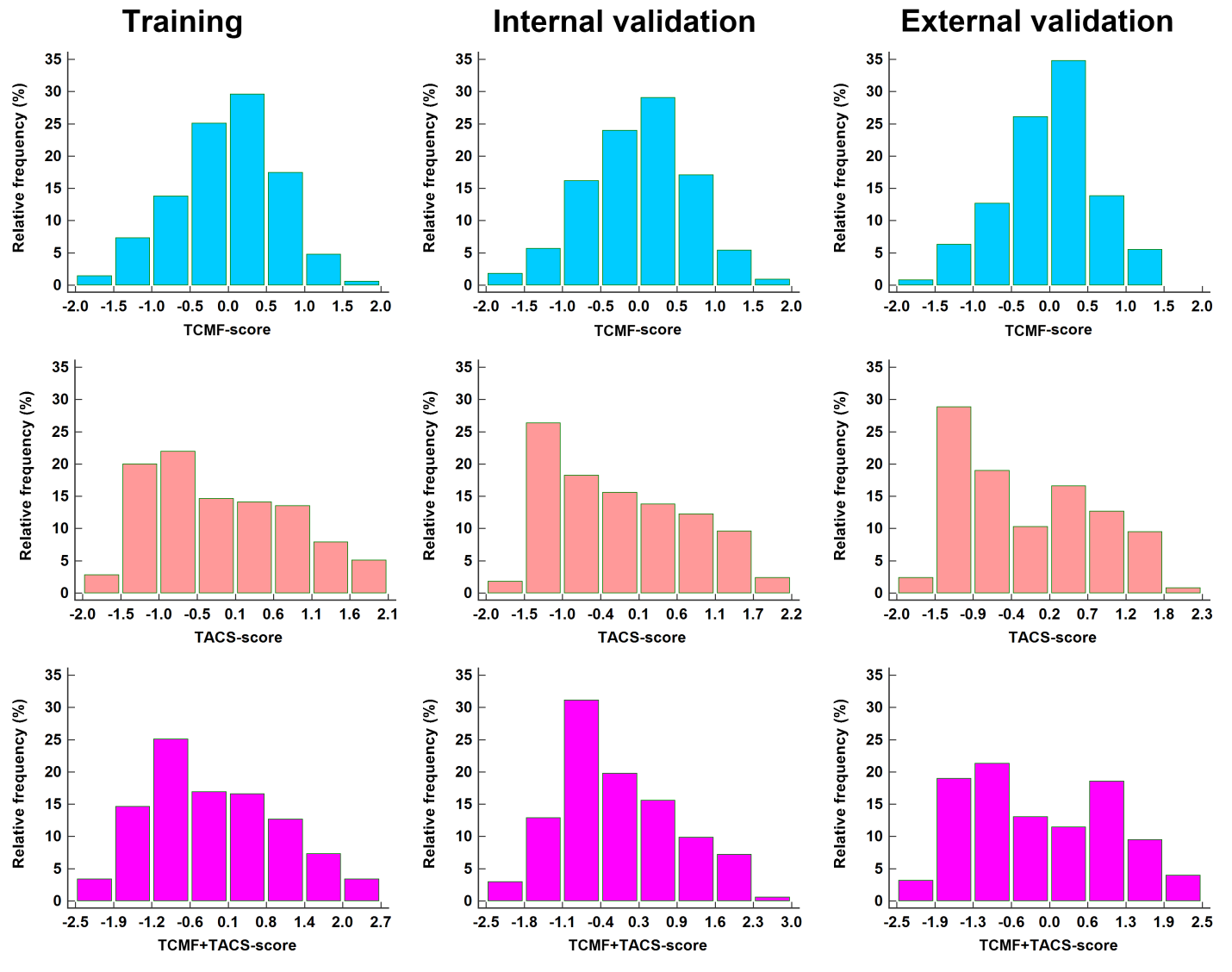


**Figure S2.** Distribution histogram of TCMF-score, TACS-score and TCMF+TACS-score in the training, internal validation and external validation cohorts.


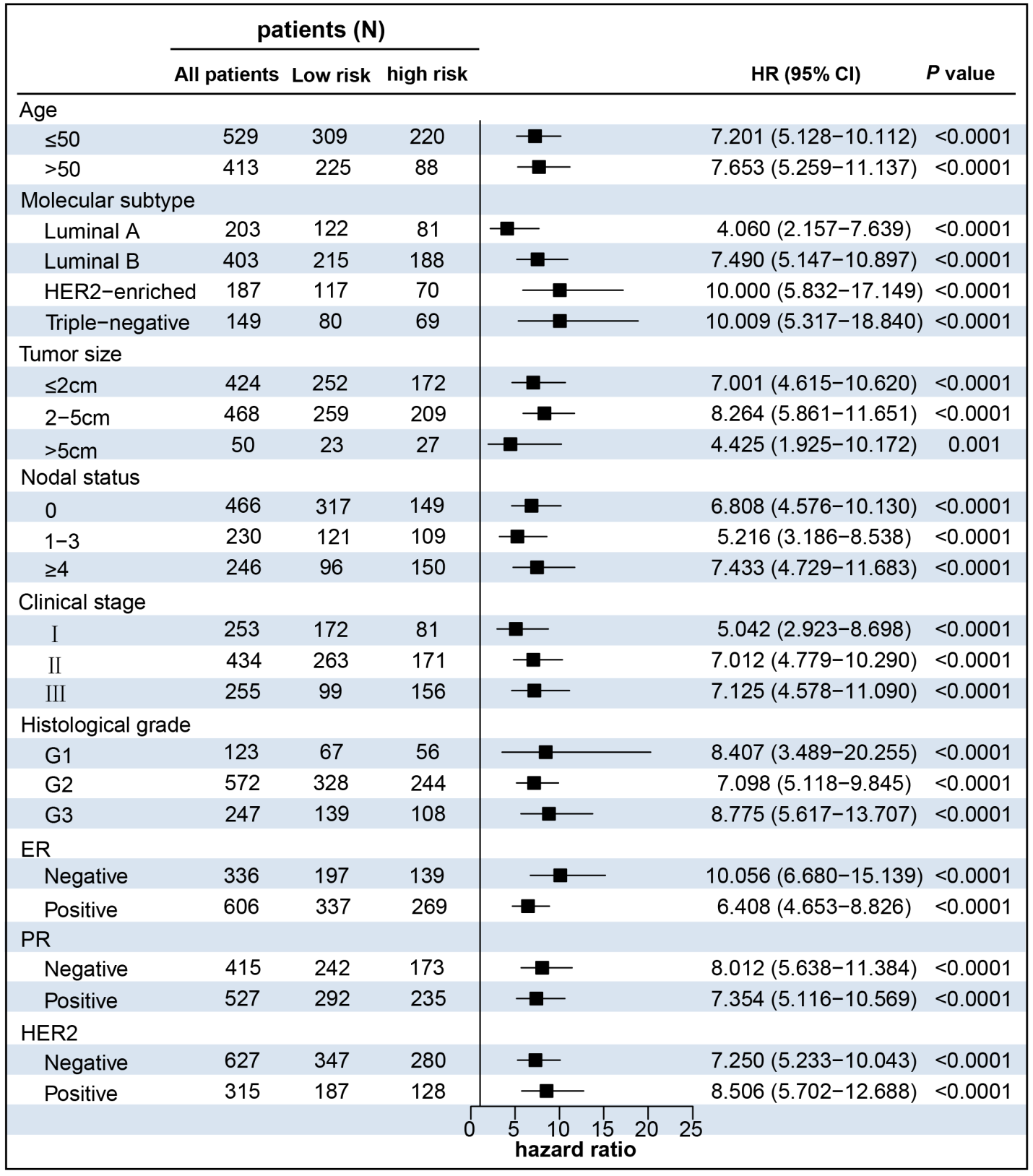


**Figure S3.** Hazard ratio of 5-year recurrence for all patients according to the TCMF+TACS-score classifier in diﬀerent subgroups stratified by clinical parameters.


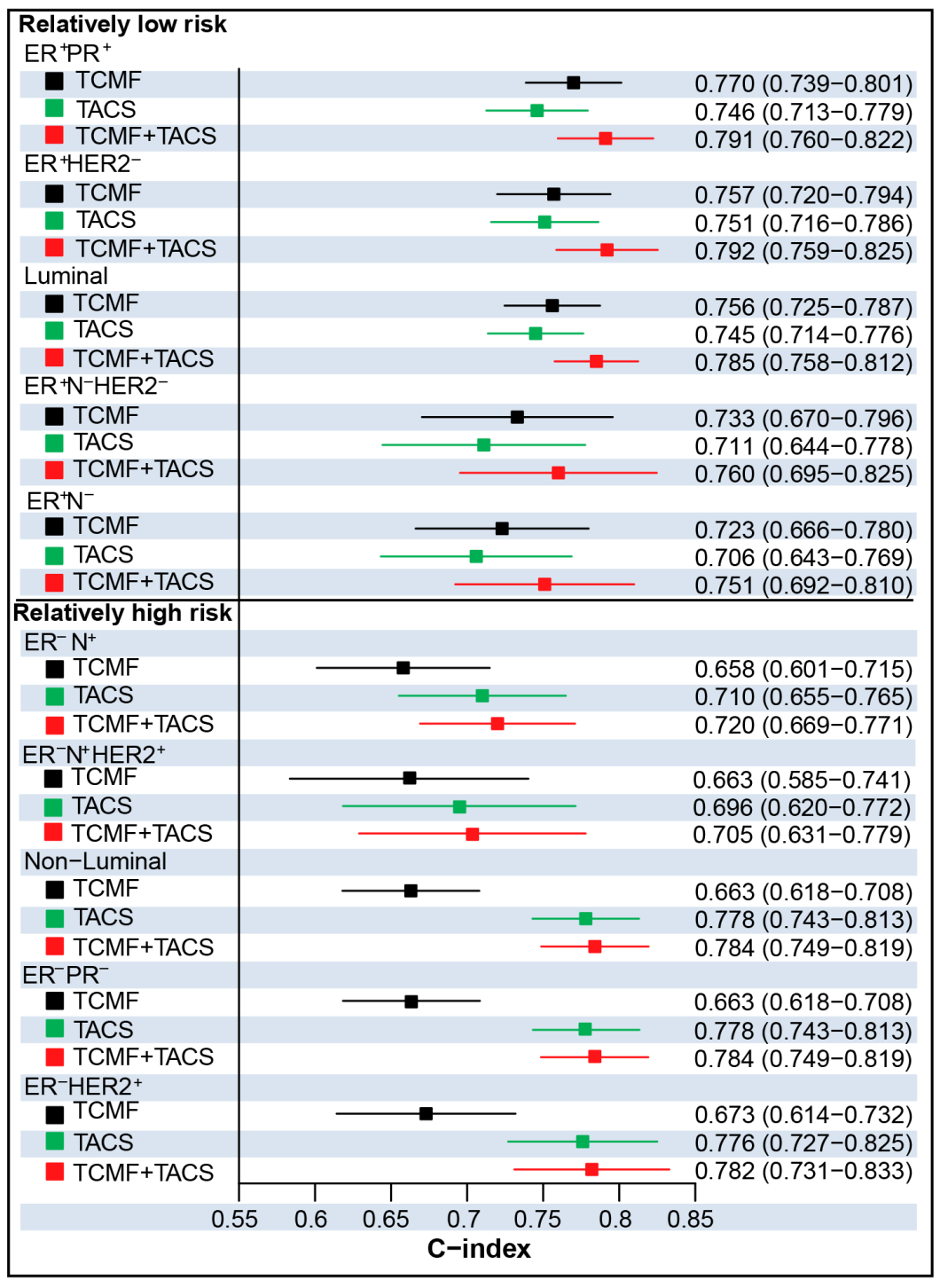


**Figure S4.** Forest plot shows the concordance index (*C*-index) of different prediction models (TCMF, TACS, and TCMF+TACS) in the different risk combination patients. N^+^/ N^–^, lymph node positive/negative; ER^+^/ ER^–^, estrogen receptors positive/negative; PR^+^/ PR^–^, progesterone receptors positive/negative; HER2^+^/ HER2^–^, HER2 positive/negative; Luminal, Luminal A/B; Non-luminal, HER2-enriched/Triple negative.


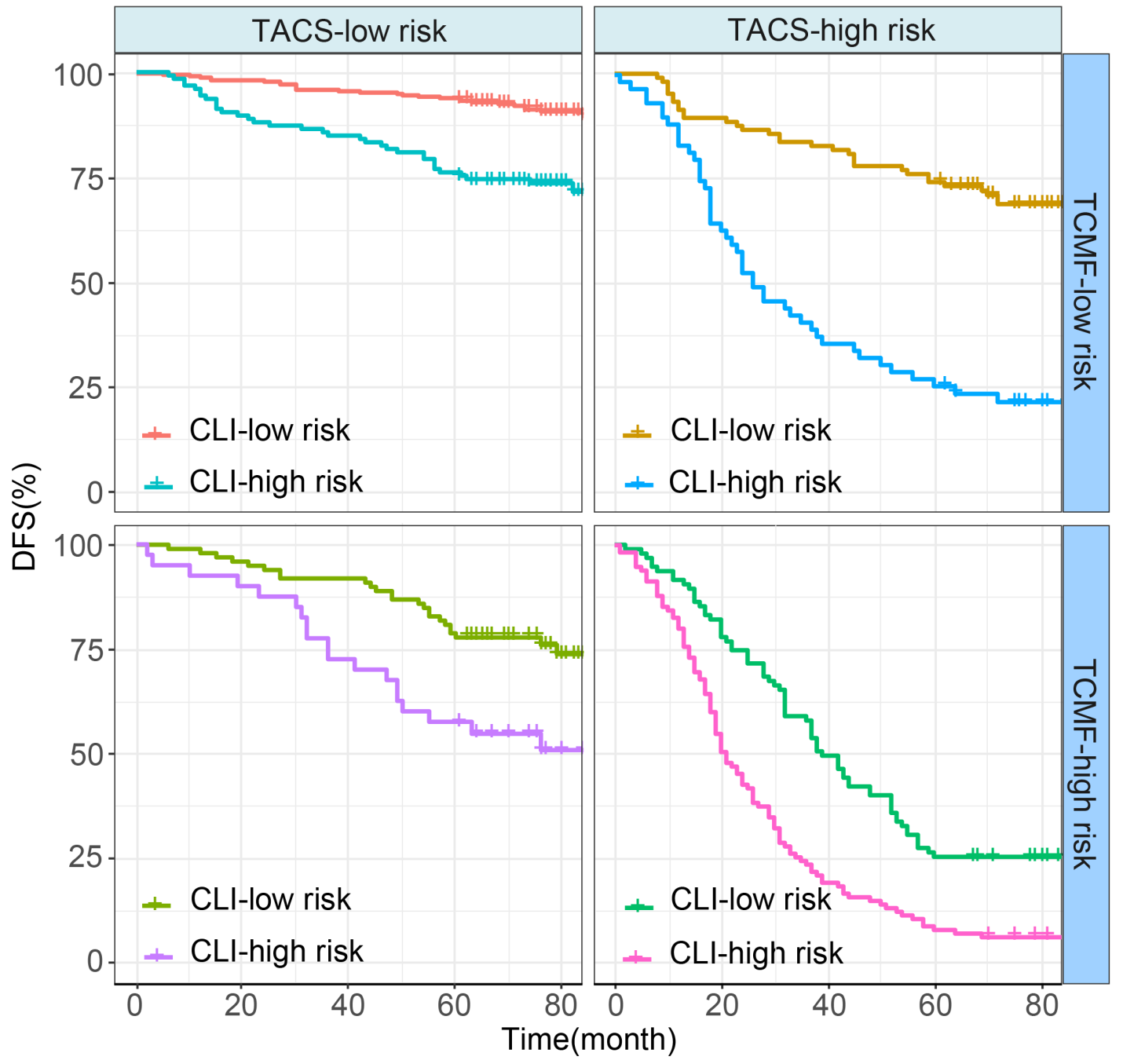


**Figure S5.** Kaplan-Meier survival curves for comparing the risk stratification ability of the CLI-score under four different situations (TACS-low risk and TCMF-low risk, TACS-high risk and TCMF-low risk, TACS-low risk and TCMF-high risk, TACS-high risk and TCMF-high risk). CLI-low risk and -high risk are the low and high risk patients classified by the CLI-score. DFS (y-axis): disease-free survival.


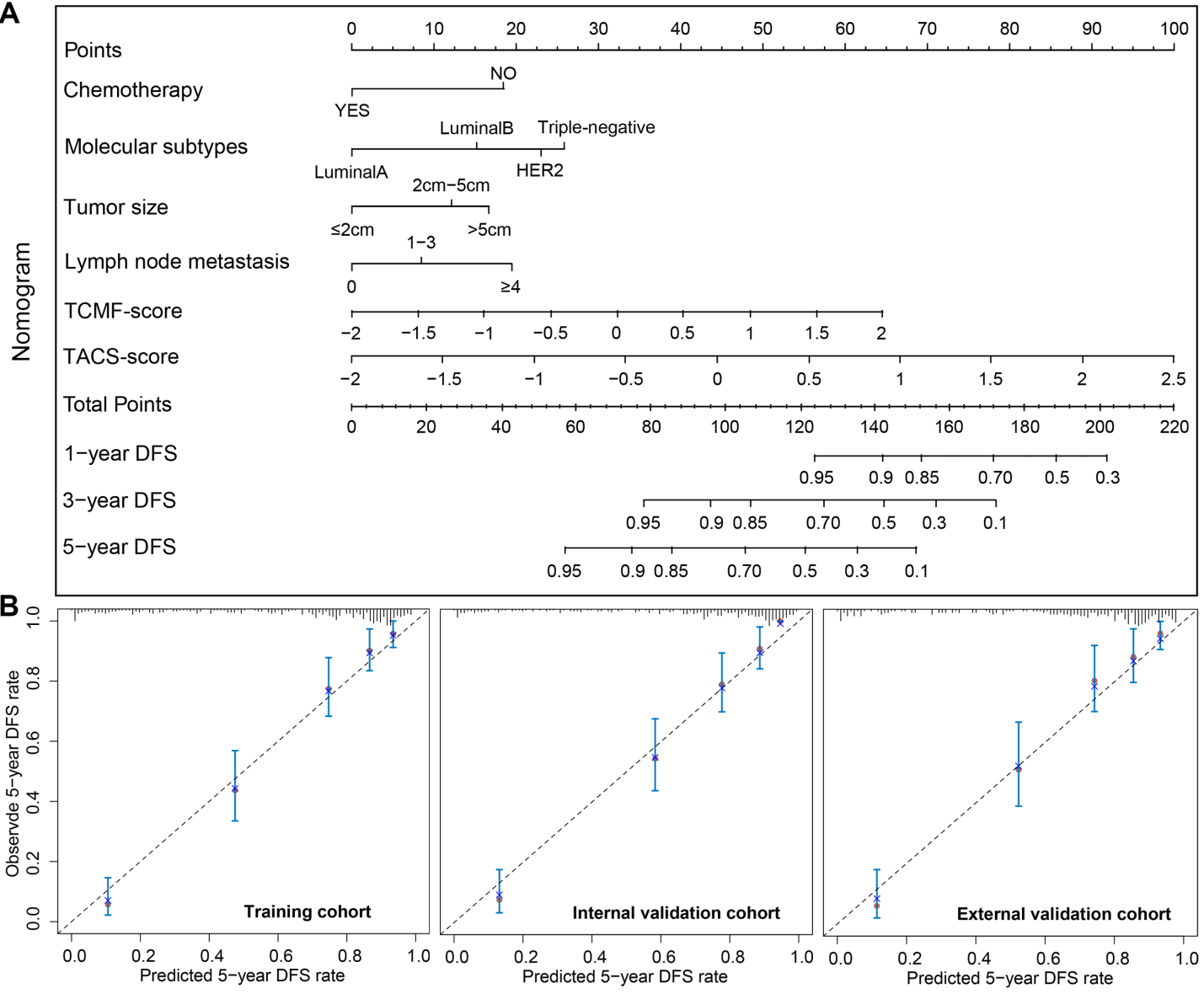


**Figure S6.** (A) Nomogram combining the TACS-score, TCMF-score, molecular subtype, tumor size, nodal status and chemotherapy from the training cohort to predict the 1-, 3-, and 5-year DFS of patients after surgery. (B) Calibration curves of the nomogram to predict 5-year DFS rate in training, internal validation, and external validation cohorts.


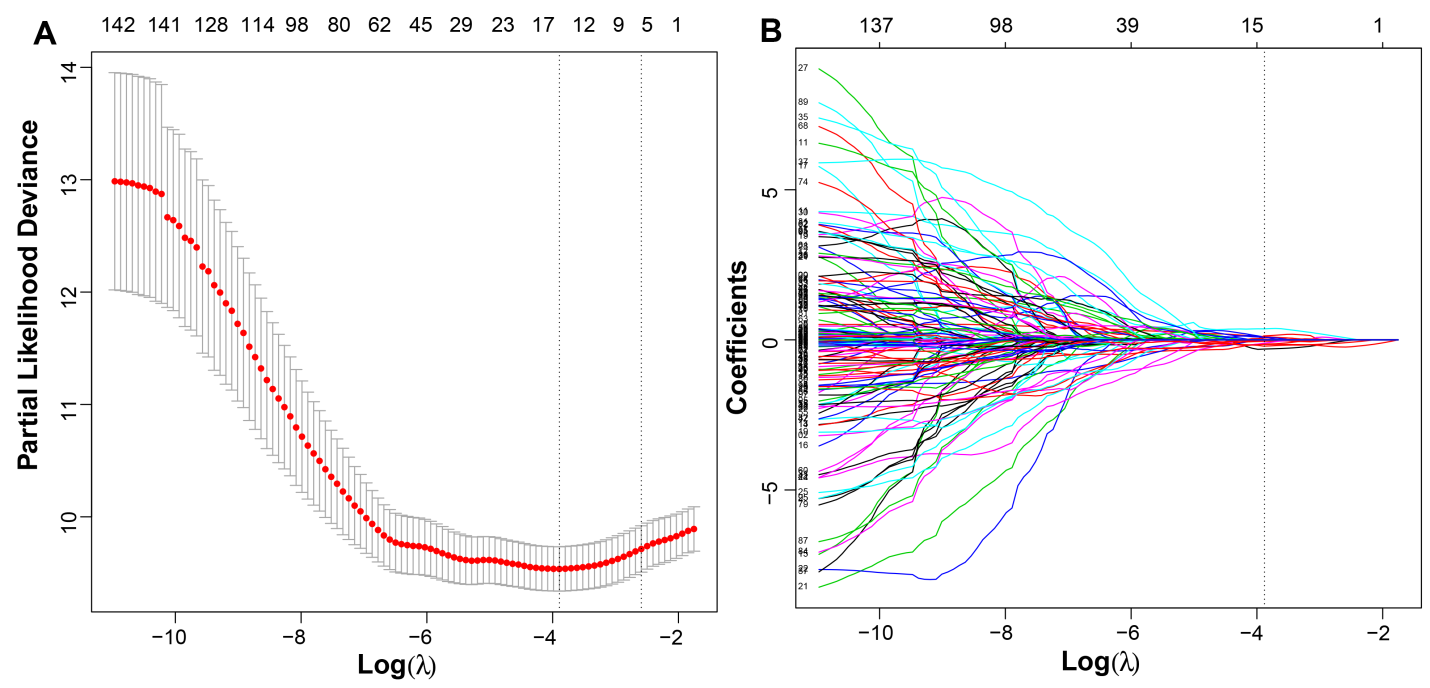


**Figure S7.** TCMF selection using LASSO cox regression analysis. (A) Plot of partial likelihood deviance for the 142 TCMF in the training cohort. Ten-fold cross-validation via minimum criteria was used for tuning the parameter (lambda) in the lasso model. The left dotted vertical line is at the optimal lambda value by using the minimum criteria, and the right line is at the optimal lambda value by using one standard error of the minimum criteria (the 1-SE criteria). (B) LASSO coefficient profiles of the 142 features. A dotted vertical line is drawn at the value selected using ten-fold cross-validation, where the optimal lambda results in 14 nonzero coefficients.


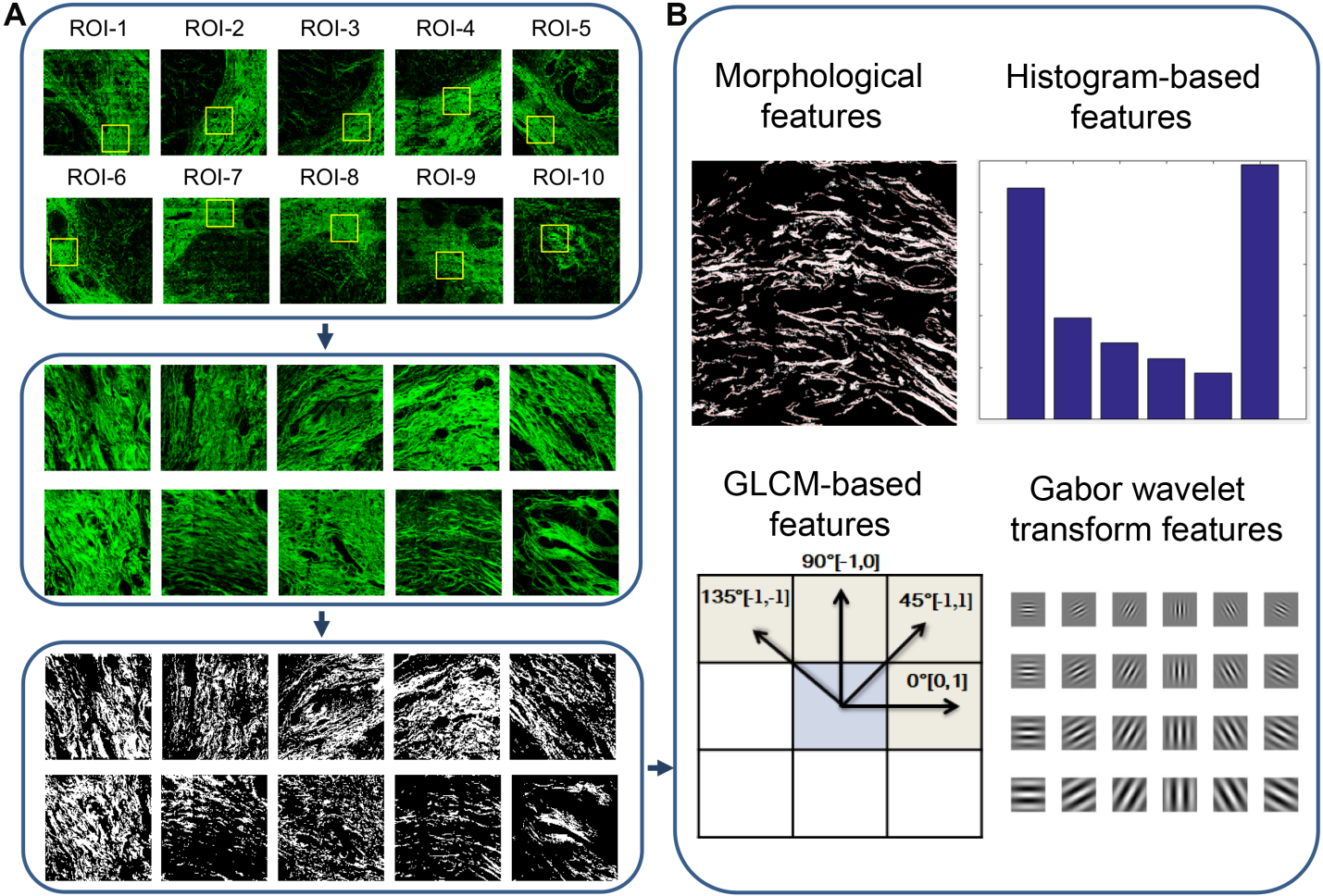


**Figure S8.** Schematic of extracting TACS corresponding microscopy features (TCMF). (A) A region of interest with a field of view of 150μm × 150μm is intercepted from the large-scale TACS, and then the intercepted SHG image is divided into collagen fibers and background. (B) Four types of TCMF are extracted, including morphological features, histogram-based features, GLCM-based features and Gabor wavelet transform features.


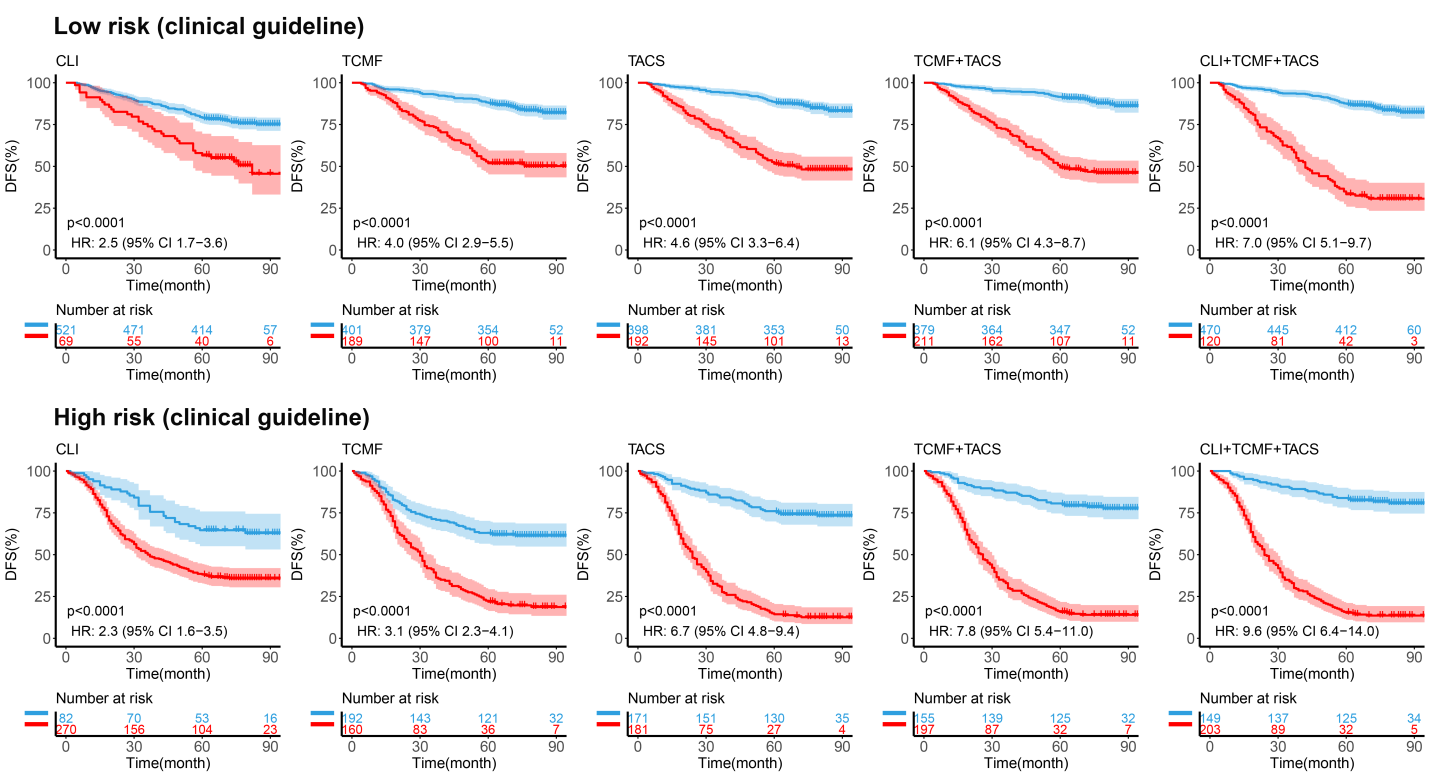


**Figure S9.** Kaplan-Meier curves of DFS according to the CLI, TCMF, TACS, TCMF+TACS, and CLI+TCMF+TACS models for the low risk and high risk patients classified by the treatment guideline. Blue lines show subjects with low risk group, and red lines show subjects with high risk group. DFS: disease-free survival, HR: hazard ratio.


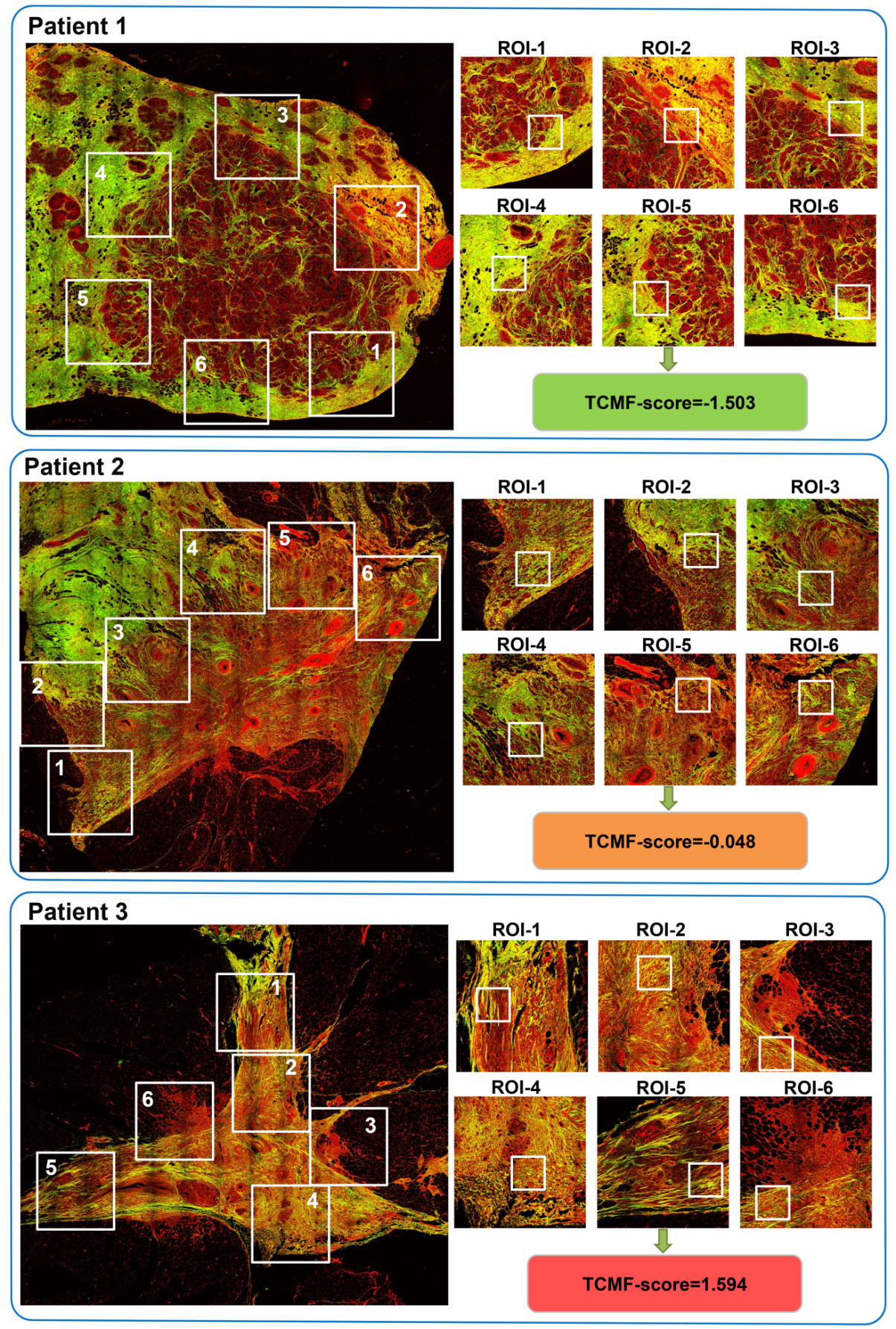


**Figure S10.** Representative MPM images of three patients to illustrate the extraction of TCMF. Patient 1: low TCFM-score; patient 2: medium TCFM-score; and patient 3: high TCFM-score.

**Supplementary Tables**

| **Table S1. Univariate and multivariate Cox proportional hazards regression analyses of the association of variables with DFS in the training cohort.** | | | | | | | | | | | | | | |
| --- | --- | --- | --- | --- | --- | --- | --- | --- | --- | --- | --- | --- | --- | --- |
| **Variable** | **Univariate analysis** | | | | | |  | | **Multivariate analysis** | | | | | |
|  | **HR** | **(95%CI)** | | | ***P* Value** | |  | | **HR** | **(95%CI)** | | | ***P* Value** | |
| **Age** |  |  |  |  | |  | |  | | |  |  | |  |
| >50 vs ≤50 | 1.156 | 0.831 | 1.610 | 0.389 | |  | | NA | | |  |  | | NA |
| **Molecular subtype** |  |  |  |  | |  | |  | | |  |  | |  |
| Luminal B vs Luminal A | 1.995 | 1.218 | 3.269 | 0.006 | |  | | 1.840 | | | 1.114 | 3.039 | | 0.017 |
| HER2-enriched vs Luminal A | 1.754 | 0.974 | 3.158 | 0.061 | |  | | 2.517 | | | 1.382 | 4.584 | | 0.003 |
| Triple-negative vs Luminal A | 1.691 | 0.917 | 3.121 | 0.093 | |  | | 2.810 | | | 1.498 | 5.270 | | 0.001 |
| **Tumor size** |  |  |  |  | |  | |  | | |  |  | |  |
| 2-5cm vs ≤2cm | 1.616 | 1.120 | 2.331 | 0.010 | |  | | 1.618 | | | 1.104 | 2.372 | | 0.014 |
| ≥5cm vs ≤2cm | 3.364 | 1.868 | 6.059 | 5.301E-05 | |  | | 1.976 | | | 1.053 | 3.709 | | 0.034 |
| **Nodal status** |  |  |  |  | |  | |  | | |  |  | |  |
| 1-3 vs 0 | 1.518 | 0.951 | 2.425 | 0.080 | |  | | 1.405 | | | 0.86 | 2.294 | | 0.175 |
| ≥4 vs 0 | 3.430 | 2.355 | 4.997 | 1.343E-10 | |  | | 2.177 | | | 1.417 | 3.344 | | 3.81E-04 |
| **Clinical stage** |  |  |  |  | |  | |  | | |  |  | |  |
| ⅡvsⅠ | 1.588 | 0.956 | 2.636 | 0.074 | |  | | NA | | |  |  | | NA |
| ⅢvsⅠ | 4.227 | 2.585 | 6.910 | 9.067E-09 | |  | | NA | | |  |  | | NA |
| **Histological grade** |  |  |  |  | |  | |  | | |  |  | |  |
| G2 vs G1 | 1.249 | 0.755 | 2.065 | 0.386 | |  | | NA | | |  |  | | NA |
| G3 vs G1 | 1.520 | 0.890 | 2.595 | 0.125 | |  | | NA | | |  |  | | NA |
| **Chemotherapy** |  |  |  |  | |  | |  | | |  |  | |  |
| No vs Yes | 1.875 | 1.129 | 3.113 | 0.015 | |  | | 2.114 | | | 1.236 | 3.614 | | 0.006 |
| **Radiation therapy** |  |  |  |  | |  | |  | | |  |  | |  |
| No vs Yes | 0.688 | 0.491 | 0.963 | 0.029 | |  | | NA | | |  |  | | NA |
| **TCMF-score** | 3.667 | 2.716 | 4.953 | 2.311E-17 | |  | | 1.911 | | | 1.407 | 2.595 | | 3.40E-05 |
| **TACS-score** | 2.890 | 2.410 | 3.466 | 2.189E-30 | |  | | 2.437 | | | 1.977 | 3.003 | | 6.55E-17 |
| Abbreviations: NA, not available. | | | | | | | | | | | | | | |

| **Table S2. Univariate and multivariate Cox proportional hazards regression analyses of the association of variables with DFS in the internal validation cohort.** | | | | | | | | | | | | | | |
| --- | --- | --- | --- | --- | --- | --- | --- | --- | --- | --- | --- | --- | --- | --- |
| **Variable** | **Univariate analysis** | | | | | |  | | **Multivariate analysis** | | | | | |
|  | **HR** | **(95%CI)** | | | ***P* Value** | |  | | **HR** | **(95%CI)** | | | ***P* Value** | |
| **Age** |  |  |  |  | |  | |  | | |  |  | |  |
| >50 vs ≤50 | 1.202 | 0.839 | 1.723 | 0.315 | |  | | NA | | |  |  | | NA |
| **Molecular subtype** |  |  |  |  | |  | |  | | |  |  | |  |
| Luminal B vs Luminal A | 2.919 | 1.442 | 5.908 | 0.003 | |  | | 2.687 | | | 1.315 | 5.492 | | 0.007 |
| HER2-enriched vs Luminal A | 2.995 | 1.412 | 6.350 | 0.004 | |  | | 2.694 | | | 1.244 | 5.832 | | 0.012 |
| Triple-negative vs Luminal A | 4.733 | 2.232 | 10.038 | 5.07E-05 | |  | | 4.472 | | | 2.061 | 9.701 | | 1.50E-04 |
| **Tumor size** |  |  |  |  | |  | |  | | |  |  | |  |
| 2-5cm vs ≤2cm | 2.175 | 1.422 | 3.325 | 3.35E-04 | |  | | 1.847 | | | 1.200 | 2.843 | | 0.005 |
| ≥5cm vs ≤2cm | 3.891 | 2.116 | 7.156 | 1.23E-05 | |  | | 2.132 | | | 1.132 | 4.014 | | 0.019 |
| **Nodal status** |  |  |  |  | |  | |  | | |  |  | |  |
| 1-3 vs 0 | 1.682 | 1.043 | 2.714 | 0.033 | |  | | 1.458 | | | 0.870 | 2.444 | | 0.152 |
| ≥4 vs 0 | 3.732 | 2.463 | 5.655 | 5.28E-10 | |  | | 2.286 | | | 1.472 | 3.551 | | 2.33E-04 |
| **Clinical stage** |  |  |  |  | |  | |  | | |  |  | |  |
| ⅡvsⅠ | 1.795 | 1.023 | 3.148 | 0.041 | |  | | NA | | |  |  | | NA |
| ⅢvsⅠ | 4.391 | 2.507 | 7.691 | 2.29E-07 | |  | | NA | | |  |  | | NA |
| **Histological grade** |  |  |  |  | |  | |  | | |  |  | |  |
| G2 vs G1 | 1.597 | 0.895 | 2.849 | 0.113 | |  | | NA | | |  |  | | NA |
| G3 vs G1 | 1.928 | 1.053 | 3.532 | 0.033 | |  | | NA | | |  |  | | NA |
| **Chemotherapy** |  |  |  |  | |  | |  | | |  |  | |  |
| No vs Yes | 1.790 | 1.042 | 3.075 | 0.035 | |  | | NA | | |  |  | | NA |
| **Radiation therapy** |  |  |  |  | |  | |  | | |  |  | |  |
| No vs Yes | 0.827 | 0.566 | 1.209 | 0.327 | |  | | NA | | |  |  | | NA |
| **TCMF -score** | 3.354 | 2.470 | 4.554 | 8.82E-15 | |  | | 2.149 | | | 1.564 | 2.954 | | 2.43E-06 |
| **TACS-score** | 2.738 | 2.233 | 3.356 | 3.14E-22 | |  | | 2.363 | | | 1.910 | 2.923 | | 2.43E-15 |
| Abbreviations: NA, not available. |  |  |  |  | |  | |  | | |  |  | |  |

| **Table S3. Univariate and multivariate Cox proportional hazards regression analyses of the association of variables with DFS in the external validation cohort.** | | | | | | | | | | | | | | |
| --- | --- | --- | --- | --- | --- | --- | --- | --- | --- | --- | --- | --- | --- | --- |
| **Variable** | **Univariate analysis** | | | | | |  | | **Multivariate analysis** | | | | | |
|  | **HR** | **(95%CI)** | | | ***P* Value** | |  | | **HR** | **(95%CI)** | | | ***P* Value** | |
| **Age** |  |  |  |  | |  | |  | | |  |  | |  |
| >50 vs ≤50 | 1.235 | 0.834 | 1.828 | 0.292 | |  | | NA | | |  |  | | NA |
| **Molecular subtype** |  |  |  |  | |  | |  | | |  |  | |  |
| Luminal B vs Luminal A | 2.340 | 1.315 | 4.164 | 0.004 | |  | | 1.830 | | | 0.998 | 1.830 | | 0.051 |
| HER2-enriched vs Luminal A | 2.613 | 1.379 | 4.951 | 0.003 | |  | | 2.640 | | | 1.335 | 2.640 | | 0.005 |
| Triple-negative vs Luminal A | 2.936 | 1.508 | 5.714 | 0.002 | |  | | 2.526 | | | 1.237 | 2.526 | | 0.011 |
| **Tumor size** |  |  |  |  | |  | |  | | |  |  | |  |
| 2-5cm vs ≤2cm | 1.395 | 0.940 | 2.070 | 0.098 | |  | | NA | | |  |  | | NA |
| ≥5cm vs ≤2cm | 0.885 | 0.122 | 6.401 | 0.903 | |  | | NA | | |  |  | | NA |
| **Nodal status** |  |  |  |  | |  | |  | | |  |  | |  |
| 1-3 vs 0 | 1.761 | 1.066 | 2.909 | 0.027 | |  | | 1.355 | | | 0.796 | 1.355 | | 0.263 |
| ≥4 vs 0 | 3.850 | 2.387 | 6.212 | 3.29E-08 | |  | | 2.276 | | | 1.340 | 2.276 | | 0.002 |
| **Clinical stage** |  |  |  |  | |  | |  | | |  |  | |  |
| ⅡvsⅠ | 1.372 | 0.802 | 2.347 | 0.248 | |  | | NA | | |  |  | | NA |
| ⅢvsⅠ | 3.753 | 2.189 | 6.434 | 1.52E-06 | |  | | NA | | |  |  | | NA |
| **Histological grade** |  |  |  |  | |  | |  | | |  |  | |  |
| G2 vs G1 | 1.414 | 0.445 | 4.492 | 0.557 | |  | | 0.945 | | | 0.289 | 0.945 | | 0.925 |
| G3 vs G1 | 4.797 | 1.456 | 15.811 | 0.010 | |  | | 1.736 | | | 0.493 | 1.736 | | 0.391 |
| **Chemotherapy** |  |  |  |  | |  | |  | | |  |  | |  |
| No vs Yes | 0.723 | 0.365 | 1.435 | 0.354 | |  | | NA | | |  |  | | NA |
| **Radiation therapy** |  |  |  |  | |  | |  | | |  |  | |  |
| No vs Yes | 0.428 | 0.283 | 0.649 | 6.26E-05 | |  | | NA | | |  |  | | NA |
| **TCMF -score** | 4.964 | 3.344 | 7.370 | 1.90E-15 | |  | | 2.569 | | | 1.615 | 2.569 | | 6.84E-05 |
| **TACS-score** | 2.455 | 1.989 | 3.029 | 5.69E-17 | |  | | 1.804 | | | 1.401 | 2.323 | | 4.71E-06 |
| Abbreviations: NA, not available. |  |  |  |  | |  | |  | | |  |  | |  |

| **Table S4. Risk stratification ability of TCMF+TACS-score in subgroups of the three cohorts.** | | | | | | |
| --- | --- | --- | --- | --- | --- | --- |
| **Characteristics** | **Fuzhou**  **training cohort (355)** | | **Fuzhou internal**  **validation cohort (334)** | | **Harbin external**  **validation cohort (253)** | |
|  | **Low risk** | **High risk** | **Low risk** | **High risk** | **Low risk** | **High risk** |
| **Age** |  |  |  |  |  |  |
| ≤50 | 117(58.8%) | 82 (41.2%) | 115 (59.3%) | 79 (40.7%) | 77 (56.6%) | 59 (43.4%) |
| >50 | 83 (53.2%) | 73 (46.8%) | 78 (55.7%) | 62 (44.3%) | 64 (54.7%) | 53 (45.3%) |
| **Molecular subtype** |  |  |  |  |  |  |
| Luminal A | 46 (61.3%) | 29 (38.7%) | 36 (61.0%) | 23 (39.0%) | 40 (58.0%) | 29 (42.0%) |
| Luminal B | 86 (53.4%) | 75 (46.6%) | 82 (56.6%) | 63 (43.4%) | 47 (48.5%) | 50 (51.5%) |
| HER2-enriched | 39 (60.9%) | 25 (39.1%) | 44 (58.7%) | 31 (41.3%) | 34 (70.8%) | 14 (29.2%) |
| Triple-negative | 29 (52.7%) | 26 (47.3%) | 31 (56.4%) | 24 (43.6%) | 20 (51.3%) | 19 (48.7%) |
| **Tumor size** |  |  |  |  |  |  |
| ≤2cm | 90 (61.2%) | 57(38.8%) | 84 (62.7%) | 50 (37.3%) | 78 (54.5%) | 65 (45.5%) |
| 2-5cm | 101(54.0%) | 86 (46.0%) | 97 (55.7%) | 77 (44.3%) | 61 (57.0%) | 46 (43.0%) |
| >5cm | 9 (42.9%) | 12 (57.1%) | 12 (46.2%) | 14 (53.8%) | 2 (66.7%) | 1 (33.3%) |
| **Nodal status** |  |  |  |  |  |  |
| 0 | 120(66.7%) | 60 (33.3%) | 116 (68.6%) | 53 (31.4%) | 81 (69.2%) | 36 (30.8%) |
| 1-3 | 44 (59.5%) | 30 (40.5%) | 39 (48.1%) | 42 (51.9%) | 38 (50.7%) | 37 (49.3%) |
| ≥4 | 36 (35.6%) | 65 (64.4%) | 38 (45.2%) | 46 (54.8%) | 22 (36.1%) | 39 (63.9%) |
| **Clinical stage** |  |  |  |  |  |  |
| Ⅰ | 65 (67.7%) | 31 (32.3%) | 59 (71.1%) | 24 (28.9%) | 48 (64.9%) | 26 (35.1%) |
| Ⅱ | 99 (63.5%) | 57 (36.5%) | 93 (57.8%) | 68 (42.2%) | 71 (60.7%) | 46 (39.3%) |
| Ⅲ | 36 (35.0%) | 67 (65.0%) | 41 (45.6%) | 49 (54.4%) | 22 (35.5%) | 40 (64.5%) |
| **Histological grade** |  |  |  |  |  |  |
| G1 | 31 (55.4%) | 25 (44.6%) | 30 (53.6%) | 26 (46.4%) | 6 (54.5%) | 5 (45.5%) |
| G2 | 104(53.6%) | 90 (46.4%) | 103 (58.5%) | 73 (41.5%) | 121 (59.9%) | 81 (40.1%) |
| G3 | 65 (61.9%) | 40 (38.1%) | 60 (58.8%) | 42 (41.2%) | 14 (35.0%) | 26 (65.0%) |
| **ER** |  |  |  |  |  |  |
| Negative | 68 (57.1%) | 51 (42.9%) | 75 (57.7%) | 55 (42.3%) | 54 (62.1%) | 33 (37.9%) |
| Positive | 132(55.9%) | 104 (44.1%) | 118 (57.8%) | 86 (42.2%) | 87 (52.4%) | 79 (47.6%) |
| **PR** |  |  |  |  |  |  |
| Negative | 82 (53.9%) | 70 (46.1%) | 93 (60.0%) | 62 (40.0%) | 67 (62.0%) | 41 (38.0%) |
| Positive | 118(58.1%) | 85 (41.9%) | 100 (55.9%) | 79 (44.1%) | 74 (51.0%) | 71 (49.0%) |
| **HER2** |  |  |  |  |  |  |
| Negative | 132(54.3%) | 111 (45.7%) | 121 (56.8%) | 92 (43.2%) | 94 (55.0%) | 77 (45.0%) |
| Positive | 68 (60.7%) | 44 (39.3%) | 72 (59.5%) | 49 (40.5%) | 47 (57.3%) | 35 (42.7%) |
| **Chemotherapy** |  |  |  |  |  |  |
| No | 11 (39.3%) | 17 (60.7%) | 17 (60.7%) | 11 (39.3%) | 20 (74.1%) | 7 (25.9%) |
| Yes | 189(57.8%) | 138 (42.2%) | 176 (57.5%) | 130 (42.5%) | 121 (53.5%) | 105 (46.5%) |
| **Endocrine Therapy** |  |  |  |  |  |  |
| No | 75 (55.6%) | 60 (44.4%) | 84 (60.0%) | 56 (40.0%) | 78 (58.6%) | 55 (41.4%) |
| Yes | 125(56.8%) | 95 (43.2%) | 109 (56.2%) | 85 (43.8%) | 63 (52.5%) | 57 (47.5%) |
| **Radiation Therapy** |  |  |  |  |  |  |
| No | 135(57.7%) | 99 (42.3%) | 140 (60.3%) | 92 (39.7%) | 118 (60.5%) | 77 (39.5%) |
| Yes | 65 (53.7%) | 56 (46.3%) | 53 (52.0%) | 49 (48.0%) | 23 (39.7%) | 35 (60.3%) |
| **Targeted Therapy** |  |  |  |  |  |  |
| No | 184(55.6%) | 147 (44.4%) | 176 (56.6%) | 135 (43.4%) | 126 (55.3%) | 102 (44.7%) |
| Yes | 16 (66.7%) | 8 (33.3%) | 17 (73.9%) | 6 (26.1%) | 15 (60.0%) | 10 (40.0%) |
| NOTE: ER, Estrogen Receptor; PR, Progesterone Receptor. | | | | | | |

| **Table S5.** **Risk stratification ability of the five prediction models in three cohorts.** | | | | | | | |
| --- | --- | --- | --- | --- | --- | --- | --- |
| **model** | **Cohort** | **5-yr DFS (607 64.4%)** | **Predicted low risk** | **Predicted high risk** | **HR** | **95% CI** | ***P* value** |
|  | **T** | 222 (62.5%) | 215 (60.6%) | 140 (39.4%) | 3.76 | 2.66-5.32 | 5.56E-14 |
| **CLI** | **I-V** | 222 (66.5%) | 214 (64.1%) | 120 (35.9%) | 3.30 | 2.30-4.75 | 1.17E-10 |
|  | **E-V** | 163 (64.4%) | 174 (68.8%) | 79 (31.2%) | 2.93 | 1.98-4.34 | 9.14E-08 |
|  | **T** | 222 (62.5%) | 220 (62.0%) | 135 (38.0%) | 4.36 | 3.08-6.17 | 1.14E-16 |
| **TCMF-score** | **I-V** | 222 (66.5%) | 206 (61.7%) | 128 (38.3%) | 3.52 | 2.42-5.11 | 3.90E-11 |
|  | **E-V** | 163 (64.4%) | 167 (66.0%) | 86 (34.0%) | 3.37 | 2.26-5.01 | 2.07E-09 |
|  | **T** | 222 (62.5%) | 214 (60.3%) | 141 (39.7%) | 8.25 | 5.58-12.19 | 3.66E-26 |
| **TACS-score** | **I-V** | 222 (66.5%) | 205 (61.4%) | 129 (38.6%) | 4.86 | 3.31-7.14 | 7.95E-16 |
|  | **E-V** | 163 (64.4%) | 150 (59.3%) | 103 (40.7%) | 4.98 | 3.25-7.64 | 1.64E-13 |
|  | **T** | 222 (62.5%) | 200 (56.3%) | 155 (43.7%) | 10.17 | 6.57-15.73 | 2.29E-25 |
| **TCMF +TACS** | **I-V** | 222 (66.5%) | 193 (57.8%) | 141 (42.2%) | 7.13 | 4.63-10.98 | 4.53E-19 |
|  | **E-V** | 163 (64.4%) | 141 (55.7%) | 112(44.3%) | 5.24 | 3.36-8.17 | 2.83E-13 |
|  | **T** | 222 (62.5%) | 227 (63.9%) | 128 (36.1%) | 12.23 | 8.17-18.30 | 5.01E-34 |
| **CLI+ TCMF +TACS** | **I-V** | 222 (66.5%) | 220 (65.9%) | 114 (34.1%) | 8.73 | 5.83-13.06 | 6.69E-26 |
|  | **E-V** | 163 (64.4%) | 172 (68.0%) | 81(32.0%) | 6.92 | 4.57-10.47 | 5.62E-20 |
| NOTE: T Training cohort (n = 355), I-V Internal validation cohort (n = 334), E**–**V External validation cohort (n = 253). | | | | | | | |

| **Table S6. Prognosis performance of the five models at 5-year DFS in patients stratified by the combination of different risk factors.** | | | | | | | | | | |
| --- | --- | --- | --- | --- | --- | --- | --- | --- | --- | --- |
| **Variable** | **CLI** | | **TCMF-score** | | **TACS-score** | | **TCMF +TACS** | | **CLI+ TCMF +TACS** | |
|  | **HR** | **AUC** | **HR** | **AUC** | **HR** | **AUC** | **HR** | **AUC** | **HR** | **AUC** |
| **Luminal** | 2.989 | 0.721 | 5.174 | 0.833 | 5.187 | 0.811 | 6.408 | 0.866 | 8.350 | 0.889 |
| **ER^+^ & N^–^** | 3.594 | 0.639 | 3.768 | 0.805 | 3.959 | 0.772 | 5.222 | 0.840 | 7.871 | 0.879 |
| **ER^+^ & HER2^–^** | 3.355 | 0.727 | 5.186 | 0.828 | 4.807 | 0.808 | 6.521 | 0.862 | 8.653 | 0.890 |
| **ER^+^ & N^–^ & HER2^–^** | 4.207 | 0.663 | 3.772 | 0.816 | 3.626 | 0.781 | 5.614 | 0.850 | 7.818 | 0.889 |
| **ER^+^ & PR^+^** | 3.116 | 0.721 | 6.026 | 0.844 | 5.178 | 0.807 | 7.354 | 0.865 | 8.698 | 0.891 |
| **Non-Luminal** | 3.820 | 0.752 | 2.495 | 0.709 | 7.722 | 0.864 | 10.056 | 0.868 | 10.508 | 0.906 |
| **ER^–^ & N^+^** | 3.757 | 0.783 | 2.264 | 0.746 | 5.356 | 0.849 | 7.590 | 0.861 | 9.701 | 0.900 |
| **ER^–^ & HER2^+^** | 4.386 | 0.769 | 2.541 | 0.719 | 9.151 | 0.862 | 10.000 | 0.868 | 11.716 | 0.917 |
| **ER^–^ & N^+^ & HER2^+^** | 5.856 | 0.834 | 2.259 | 0.758 | 5.080 | 0.821 | 5.842 | 0.835 | 8.151 | 0.894 |
| **ER^–^ & PR^–^** | 3.820 | 0.752 | 2.495 | 0.709 | 7.722 | 0.864 | 10.056 | 0.868 | 10.508 | 0.906 |
| NOTE: Luminal, Luminal A / Luminal B; Non-Luminal, HER2-enriched / Triple Negative; ER/PR^+^, ER/PR positive; ER/PR^–^, ER/PR negative; N^+^, lymph node positive; N^–^, lymph node negative, HER2^–^, HER2 positive; HER2^–^, HER2 negative. | | | | | | | | | | |

| **Table S7. The number of likely undertreated (orange-highlighted) and overtreated (purple-highlighted) patients according to the Chinese treatment guideline and four models.** | | | | |
| --- | --- | --- | --- | --- |
| **Ground truth** | **successful 5-yr DFS (607)** | | **unsuccessful 5-yr DFS (335)** | |
| **Guideline** | 451 | 156 (reference) | 139(reference) | 196 |
| **CLI model** | 465 | 142 (-14) | 138 (-1) | 197 |
| **TCMF model** | 474 | 133 (-23) | 119 (-20) | 216 |
| **TACS model** | 482 | 125 (-31) | 87 (-52) | 248 |
| **CLI+TCMF+TACS model** | 536 | 71 (-85) | 83 (-56) | 252 |

| **Table S8. Morphological features.** | |  |
| --- | --- | --- |
| **Feature** | **Description** | |
| Collagen fiber area | The percentage of pixels belonging to collagen in the segmented image. | |
| Fiber density | The number of extracted fibers per mm^2^. | |
| Average fiber length | The mean length of the identified collagen fibers. The length of each fiber is defined as the sum of distances between neighboring vertices in the list. | |
| Average fiber width | The mean width of the identified collagen fibers. The width of each fiber is defined as the average distance between vertices in the list to their nearest background pixel. | |
| Average fiber straightness | The mean straightness of the identified collagen fibers. The straightness of each fiber is defined as the distance between the first and last vertex in the list divided by the fiber length. | |
| Fiber cross-link density | The ratio of the total number of crosslink points to the sum of lengths of all collagen fibers. | |
| Average cross-link space | The mean crosslink space of the identified collagen fibers. The crosslink space of each fiber is defined as the average distances between neighboring crosslink points. | |
| Collagen fiber orientation | The main orientation identified from the angular orientation distribution of the Fourier transformed image. | |

| **Table S9. First order intensity histogram features.** | |
| --- | --- |
| **Feature** | **Description** |
| Mean | Measures mean intensity value of a histogram. |
| Variance | Measures squared distances of each value of a histogram from the mean |
| Skewness | Measures asymmetry of a histogram. |
| Kurtosis | Measures “peakedeness” of a histogram (flatness of histogram). |
| Energy | Measures squared magnitude value of a histogram. |
| Entropy | Measures irregularity of a histogram. |

| **Table S10. Grey-level co-occurrence matrix-based features.** | | |
| --- | --- | --- |
| **Feature** | **Description** | **Formula** |
| Contrast | Uniformity of the image grayscale distribution and degree of thickness in texture. | $contrast=\sum_{i=1}^{Ng} \sum_{j=1}^{Ng} \left\vert i-j \right\vert^{2}P(i,j)$ |
| Correlation | Measurement of the gray-level linear dependence between pixels at specified positions relative to each other. | $correlation=\frac{\sum_{i=1}^{Ng} \sum_{j=1}^{Ng} ijP\left( i,j \right)-\mu_{i}(i)\mu_{j}(j)}{\sigma_{x}\left( i \right)\sigma_{y}(j)}$ |
| Energy | Sum of squares of entries in the GLCM. | $energy=\sum_{i=1}^{Ng} \sum_{j=1}^{Ng} {[P(i,j)]}^{2}$ |
| Homogeneity | Local homogeneity. | $homogeneity=\sum_{i=1}^{Ng} \sum_{j=1}^{Ng} \frac{P\left( i,j \right)}{1+\left\vert i-j \right\vert^{2}}$ |

| **Table S11. Gabor wavelet transform features (Scales and orientations of the Gabor wavelets are represented by *m* and *n* respectively).** | | |
| --- | --- | --- |
| **Feature** | **Description** | **Formula** |
| Mean | Mean of the magnitude of Gabor wavelet transform. | $mean=\iint\vert G_{mn}\left( x,y \right)\vert dxdy$ |
| Variance | Variance of the magnitude of Gabor wavelet transform. | $variance=\iint(\left\vert G_{mn}\left( x,y \right) \right\vert-mean)^{2}dxdy$ |
